# Impairment of glutamate homeostasis in the nucleus accumbens core underpins cross-sensitization to cocaine following chronic restraint stress

**DOI:** 10.3389/fphys.2022.896268

**Published:** 2022-08-26

**Authors:** María P. Avalos, Andrea S. Guzman, Constanza Garcia-Keller, Bethania Mongi-Bragato, María A. Esparza, Daiana Rigoni, Marianela A. Sanchez, Gastón D. Calfa, Flavia A. Bollati, Liliana M. Cancela

**Affiliations:** Departamento de Farmacología Otto Orsingher, Instituto de Farmacología Experimental de Córdoba (IFEC-CONICET), Facultad de Ciencias Químicas, Universidad Nacional de Córdoba, Córdoba, Argentina

**Keywords:** chronic restraint stress, ceftriaxone, cocaine, dendritic spines, GLT-1, glutamate, nucleus accumbens, sensitization

## Abstract

Though the facilitating influence of stress on drug abuse is well documented, the mechanisms underlying this interaction have yet to be fully elucidated. The present study explores the neurobiological mechanisms underpinning the sensitized response to the psychomotor-stimulating effects of cocaine following chronic restraint stress (CRS), emphasizing the differential contribution of both subcompartments of the nucleus accumbens (NA), the core (NAcore) and shell (NAshell), to this phenomenon. Adult male Wistar rats were restrained for 2 h/day for 7 days and, 2 weeks after the last stress exposure (day 21), all animals were randomly assigned to behavioral, biochemical or neurochemical tests. Our results demonstrated that the enduring CRS-induced increase in psychostimulant response to cocaine was paralleled by an increase of extracellular dopamine levels in the NAcore, but not the NAshell, greater than that observed in the non-stress group. Furthermore, we found that CRS induced an impairment of glutamate homeostasis in the NAcore, but not the NAshell. Its hallmarks were increased basal extracellular glutamate concentrations driven by a CRS-induced downregulation of GLT-1, blunted glutamate levels in response to cocaine and postsynaptic structural remodeling in pre-stressed animals. In addition, ceftriaxone, a known GLT-1 enhancer, prevented the CRS-induced GLT-1 downregulation, increased basal extracellular glutamate concentrations and changes in structural plasticity in the NAcore as well as behavioral cross-sensitization to cocaine, emphasizing the biological importance of GLT-1 in the comorbidity between chronic stress exposure and drug abuse. A future perspective concerning the paramount relevance of the stress-induced disruption of glutamate homeostasis as a vulnerability factor to the development of stress and substance use disorders during early life or adulthood of descendants is provided.

## 1 Introduction

Repeated life stress constitutes a critical environmental factor of vulnerability to developing psychiatric disorders, including drug addiction (Sinha, 2009). Epidemiological evidence points out a link between stressful events and drug abuse in humans (Clark et al., 2001; Boden et al., 2012). In animal models, the fact that different types of stressors and stress protocols are able to activate discrete neurobiological mechanisms to promote addictive behaviors (Pacchioni et al., 2007; Boyson et al., 2011, 2014; Esparza et al., 2012; Leão et al., 2012; Garcia-Keller et al., 2013, 2016; Lo Iacono et al., 2018; Bertagna et al., 2021; Rigoni et al., 2021; Avalos et al., 2022) and trigger relapse to drug-seeking behavior even after long periods of abstinence (Gysling, 2012; De Giovanni et al., 2016; Mantsch et al., 2016; Guzman et al., 2021) suggests that stress and addictive drugs impact on common neurobiological mechanisms.

The main explanation of the comorbidity between stress and substance use disorders (SUDs) has been the proactive effect of stress on the drug-induced activation of dopaminergic inputs to the nucleus accumbens (NA) subcompartments, the core (NAcore) and shell (NAshell), which are differentially involved in addictive behaviors (Cadoni and Di Chiara, 1999; Cadoni et al., 2000; Giorgi et al., 2005; Pacchioni et al., 2007; Garcia-Keller et al., 2013).

Glutamate transmission has also been considered a critical hub for the effects of stress on behavior. An altered glutamate homeostasis in the NAcore was related to stress-induced addictive behaviors associated with cocaine administration (Esparza et al., 2012; Garcia-Keller et al., 2013, 2016; Rigoni et al., 2021; Avalos et al., 2022). These alterations recapitulate, in part, the glutamatergic mechanisms triggered by repeated exposure to psychostimulants (Pierce et al., 1996; Knackstedt et al., 2010; Wang et al., 2017; Prieto et al., 2020). Even more, the synaptic release of glutamate in concert with the release and clearance of glutamate through glial mechanisms (i.e., the cystine-glutamate antiporter and the major glutamate transporter, GLT-1) were found impaired in the NAcore after chronic administration of a wide variety of addictive drugs (Kalivas, 2009). All these persistent alterations thought to underlie addictive behaviors have been related to enduring adaptations in medium spiny neurons (MSNs) (Kalivas, 2009). Indeed, structural remodeling of dendritic spines has been linked to behavioral adaptations induced by motivationally relevant stimuli, such as drug administration and exposure to stress (Kalivas, 2009; Golden and Russo, 2012; Garcia-Keller et al., 2016; Fox et al., 2020; Rigoni et al., 2021; Avalos et al., 2022).

The enduring effects of acute restraint stress (ARS) on cocaine-evoked dopamine and/or glutamate release, and the disruption of glutamate homeostasis in the NA underlying the potentiation of the reinforcing and psychomotor-stimulating effects of amphetamine and cocaine have been well described by our laboratory (Pacchioni et al., 2007; Garcia-Keller et al., 2013, 2016). While an acute traumatic event may be critically involved in the emergence of a neuropathological phenotype, repeated stressful stimuli, that occur on a daily basis, engender a devastating effect at a physiological level that favors the development of mental illnesses across the lifespan or even as a vulnerability factor with a transgenerational outcome (Rodgers and Bale, 2015; Rodgers et al., 2015; Yuan et al., 2016). Comparative studies concerning acute and chronic stress have demonstrated, in part, contrasting effects on neurochemical, physiological, immunological and behavioral responses (Cancela et al., 1991; Basso et al., 1994; Capriles and Cancela, 1999; McEween, 2004; Holly and Miczek, 2016). On the other hand, studies have reported habituation of several of these responses after the repeated stimulation of stress-responsive brain regions through homotypic stressful stimuli (Capriles and Cancela, 1999; Moghaddam, 2002; Patel and Hillard, 2008). Given that habituation does not appear to be a universal consequence of exposure to repeated and predictable stressors and that the long-term effect of chronic stress on accumbal glutamate homeostasis are still mostly unknown, there is a compelling pathophysiological need to focus the research on determining whether or not CRS has comparable effects to our previous data published after ARS (Garcia-Keller et al., 2013, 2016).

The main goal of this study is to address the lasting cross-sensitized effects of cocaine induced by chronic restraint stress (CRS) on dopamine transmission and glutamate homeostasis in the NAcore and NAshell. We employed a cross-sensitization model between CRS and cocaine, and 2 weeks after the last stress exposure (day 21), we assessed: 1) behavioral sensitization to cocaine, 2) *in vivo* microdialysis of cocaine-induced glutamate and dopamine release, 3) GLT-1 by western blotting, 4) structural plasticity, 5) *in vivo* no-net-flux, to determine basal extracellular glutamate concentrations, and 6) the effects of ceftriaxone, a known GLT-1 enhancer, on CRS-induced alterations of GLT-1, basal extracellular glutamate concentrations, dendritic spines morphology and behavioral cross-sensitization to cocaine.

## 2 Materials and methods

### 2.1 Animals

Adult male Wistar rats (280–320 g) were housed and bred in cages of 12 × 30 × 50 cm in the Facultad de Ciencias Químicas vivarium. They were maintained at 22°C under a 12:12 h light/dark cycle, with free access to food and water. At the beginning of the treatment, the animals were approximately 2 months old (±1 week). All rats were tested during the light cycle. All procedures were approved by the Animal Care Committee of the Facultad de Ciencias Químicas, Universidad Nacional de Córdoba, Argentina, which is consistent with the NIH Guide for the Care and Use of Laboratory Animals.

### 2.2 Drugs

Cocaine hydrochloride (Verardo y Cia, Buenos Aires, Argentina) and ceftriaxone (Investi, Buenos aires, Argentina) were diluted in sterile saline solution (NaCl 0.9%).

### 2.3 Stress protocol and experimental design

After 5 days of acclimatization to the housing facility, animals were randomly assigned to CRS and Non-CRS groups. The chronic stress group was restrained daily for 2 h (anytime between 10:00 and 14:00 h) for 7 days, while non-stressed animals were left undisturbed in their home cages. The Plexiglas cylindrical restraint devices contained slots to enable breathing and the tails of the rats emerged from the rear. Stressed animals appeared healthy as shown by their coat texture and there was no difference in body weight with non-stressed rats. Two weeks after the last stress session (day 21), animals were randomly assigned to the behavioral, biochemical or neurochemical studies. The experimental design is detailed in each Figure.

### 2.4 Ceftriaxone treatment

On day 17 after the first stress exposure, all stressed and non-stressed animals were randomly assigned to vehicle (saline solution, i. p.) or ceftriaxone (200 mg/kg, i. p.) groups. The treatment consisted of one daily injection of ceftriaxone or vehicle from day 17 through day 21, as shown in Figures 4–6. On day 21, all animals received the last dose at least 2 h before any experimental procedure.

### 2.5 Locomotor activity

On day 21, locomotor activity was tested in pre-stressed or non-stressed animals to detect behavioral sensitization. The apparatus consisted of eight rectangular cages (30.5 × 19.5 × 46.5 cm) equipped with two parallel infrared photocell beams located 3 cm above the floor. Interruption of either beam resulted in a photocell count. Rats were placed individually in the cage and interruptions of the beam were monitored, with counts at 10-min intervals. Animals were first exposed to an adaptation period of 60 min. Then, after the saline injection, photocell counts were measured for 60 min, followed by the cocaine challenge (15 mg/kg, i. p.) and another 120 min of monitoring. The cocaine dose was chosen based on a previous dose-response curve performed in the laboratory (Garcia-Keller et al., 2013).

### 2.6 Probe construction and stereotaxic surgery

Microdialysis probes were manufactured in our laboratory following Di Chiara et al. (1993) with minor modifications. On day 20 after the first stress session, microdialysis probes were unilaterally implanted through stereotaxic surgery in the NAcore (AP = +1.4; ML = +1.6, DV = −7.8) or NAshell (AP = +1.4; ML = +0.8, DV = −7.8) according to coordinates from Paxinos and Watson (2007). For more details, see Supplementary Material.

### 2.7 Microdialysis procedure and neurotransmitter determination

The day following surgery (day 21), the dialysis membrane was perfused with Ringer’s solution at a constant flow rate of 1 μl/min. Samples of the dialysate were collected every 30 min in freely-moving animals. Four baseline samples of dopamine or glutamate were collected. Later, all animals received a saline i. p. injection and samples were collected for 120 min. Subsequently, the same rats received cocaine (15 mg/kg) i. p. injections and, finally, dialysis samples were collected for 150 additional min. The dopamine and glutamate content of the perfusate were measured by reverse-phase High-Performance Liquid Chromatography (ESA Coulochem, Chelmsford, MA, United States). Changes in accumbal extracellular dopamine or glutamate levels were reported as percent from baseline (% baseline). For more details, see Supplementary Material.

### 2.8 No-net-flux *in vivo* microdialysis

On day 21, the basal concentrations of extracellular glutamate were determined by adding D-glutamate (Sigma-Aldrich, St Louis, MO, United States) to the dialysis perfusate at 0, 2.5, 5.0 and 10.0 µM to establish the concentration at which no-net-flux of glutamate occurred across the dialysis membrane of the probe. Four 30-min dialysis samples were obtained at each concentration of glutamate, and the last three samples were averaged to determine the net flux of glutamate (for more details see Supplementary Material).

### 2.9 Histology

Microdialysis probe placement was confirmed by brain histology after finishing the microdialysis experiments (procedure presented in Supplementary Material).

### 2.10 Tissue preparation and western blotting

On day 21, stressed and non-stressed animals were decapitated 45 min after cocaine (15 mg/kg i. p.) or saline injection. The 45-min time point was chosen to correlate GLT-1 expression with the peak psychomotor sensitization intensity induced by cocaine observed in the locomotor activity test. The NAcore was dissected and the bilateral slices were pooled. Crude membrane fractions were prepared for assessing GLT-1 protein expression by western blotting. Briefly, after total protein quantification according to the Bradford method, western blotting was carried out loading 30 μg-protein per lane by 12% SDS-PAGE and then transferring to a PVDF membrane. Blocking was performed using 5% w/v BSA in T-TBS. Membranes were incubated with rabbit polyclonal anti-GLT-1 (1:1,000; catalog #3838, Cell Signaling Technology, Beverly, United States) and then incubated with peroxidase-conjugated donkey anti-rabbit IgG secondary antibody (1:4,000; Jackson Laboratories, Baltimore Pike, PA, United States). Data were normalized to β-actin (internal loading control) and to the average of the control group. For more details, see Supplementary Material.

### 2.11 DiI labeling of dendritic spines

Procedures for brain collection on day 21, neuronal staining and morphological analysis of dendritic spines were performed as previously published (Calfa et al., 2012; Giachero et al., 2015) and are detailed in the supplementary material. Briefly, two weeks after the last restraint stress session and 45 min after an acute injection of saline or cocaine (15 mg/kg i.p.), animals were deeply anesthetized with ketamine/xylazine (55 mg/kg and 11 mg/kg, respectively) before being perfused transcardially, first by ice-cold PB (0.1 M, pH = 7.4) and then fixed using ice-cold 1.5% w/v PFA (dissolved in 0.1 M PB, pH = 7.4). After the brain was fixed, brain sections (150 µm thick) were obtained and dendritic portions from NAcore or NAshell MSNs were stained with small droplets (approx. 10 µm) of a saturated solution of the lipophilic dye, DiI (Invitrogen, Thermo Fisher Scientific, United States). Z-sections from labeled dendritic segments were collected using a laser scanning confocal fluorescence microscope (Olympus FluoView FV1200 or FluoView FV300, Tokyo, Japan) with silicone immersion objective lens (UPlanSApo60x/1.3). Dendritic projections were quantified manually using ImageJ software (NIH, Bethesda, United States). Spine types were classified as thin, stubby or mushroom-shaped dendritic spines. The total number and the number of each type of dendritic spine normalized to 10 µm of the dendritic segment length were counted in single segments from the z-section projections.

### 2.12 Statistical analyses

Statistical analyses were performed using GraphPad Prism 8 (GraphPad software, La Jolla, CA, United States) or Statistica 7 (Statsoft, Inc., Tulsa, OK, United States). Behavioral, biochemical and neurochemical data were analyzed by two- or three-way ANOVA. A two-tailed unpaired *t*-test was used to evaluate the baseline as well as the effect of a cocaine i. p. injection on dopamine and glutamate release in samples obtained from the *in vivo* microdialysis. To evaluate the stimulating effect of cocaine on motor activity in each experimental group, a two-tailed unpaired *t*-test was also performed. Results of dendritic spines were analyzed by two-way Nested ANOVA followed by one-way ANOVA to test significant variations in means among groups. The dendritic spine variables were nested within the animal variable and across the dendritic slices analyzed. Bonferroni’s *post-hoc* test was applied for multiple comparisons and *p* < 0.05 was considered statistically significant.

## 3 Results

### 3.1. CRS-induced behavioral cross-sensitization to cocaine occurred in parallel with potentiation of cocaine-induced enhancement of dopamine in the NAcore, but not the NAshell, in pre-stressed animals

**FIGURE 1:**
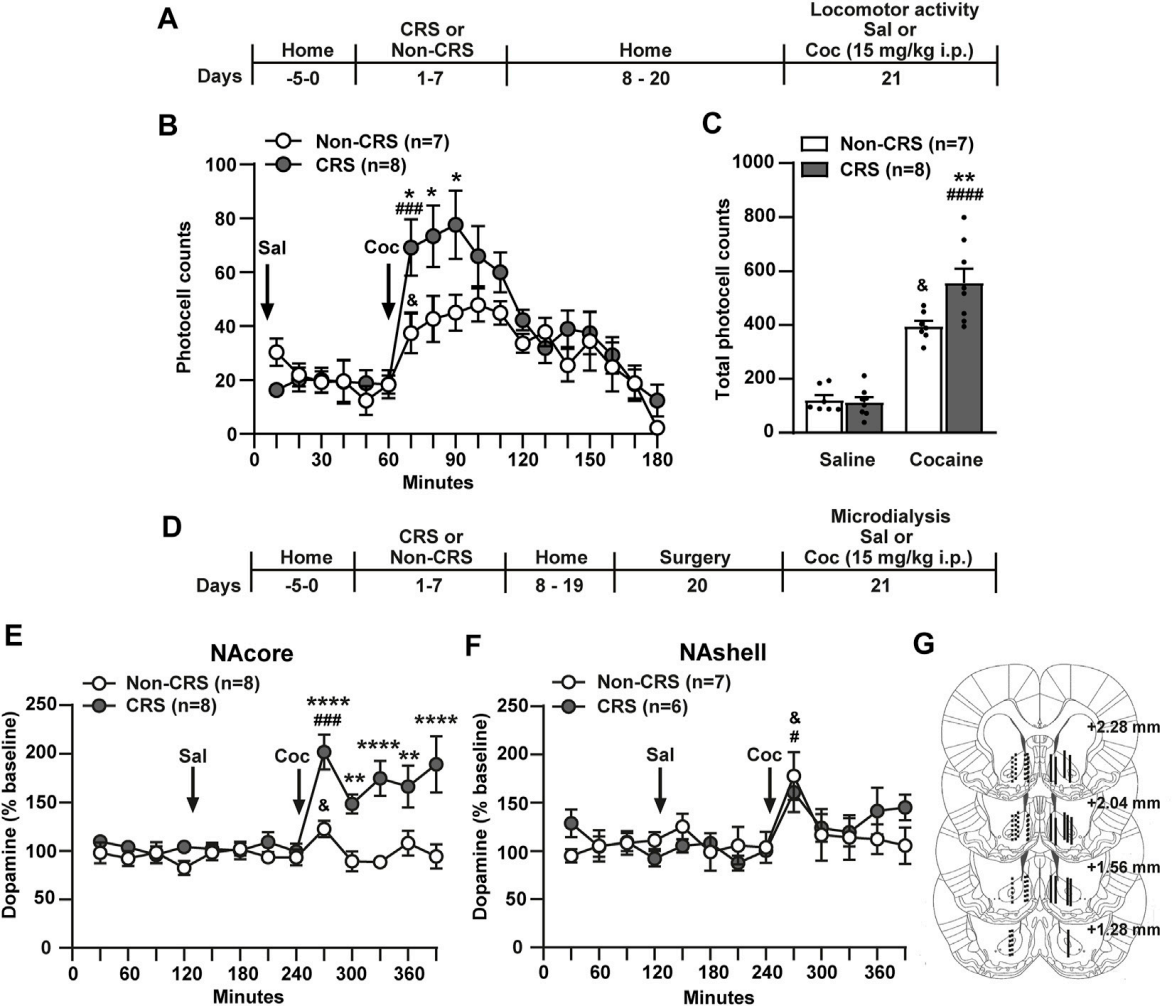
Chronic stress exposure induced behavioral and neurochemical sensitization to cocaine. **(A)** Schematic timeline of the experimental protocol. **(B)** Time course of horizontal photocell counts after saline and cocaine (15 mg/kg) i. p. injections. Time-frame from 10 to 60 min corresponds to saline administration at 0 min and time-frame from 70 to 180 min to cocaine administration. Two-way ANOVA with repeated measures over time revealed significant interaction (Non-CRS vs. CRS x time; F_(17,221)_ = 1.88, *p* < 0.05), significant effect of stress (Non-CRS vs. CRS; F_(1,13)_ = 6.05, *p* < 0.05) and time (F_(17,221)_ = 12.38, *p* < 0.0001). Bonferroni *post-hoc* test comparing Non-CRS with CRS after a cocaine challenge revealed significant differences at 70, 80 and 90 min (*p < 0.05). No differences were found comparing Non-CRS with CRS after a saline injection (n.s.). The stimulating effect of cocaine on motor activity was evaluated in each experimental group comparing results at 60 min (last data of horizontal motor activity after a saline i.p. injection) and 70 min (first data in response to a cocaine challenge). Two-tailed unpaired *t*-test revealed the psychomotor-stimulating effect of cocaine (Non-CRS: t_12_ = 2.32; ^&^ *p* < 0.05–CRS: t_14_ = 4.34; ^###^ *p* < 0.001). **(C)** Total horizontal photocell counts after a saline or cocaine (15 mg/kg) i. p. injection. Two-way ANOVA showed significant interaction (Non-CRS vs. CRS x Sal vs. Coc; F_(1,26)_ = 6.97, *p* < 0.05), significant effect of stress (Non-CRS vs. CRS; F_(1,26)_ = 5.63, *p* < 0.05) and drug (Sal vs. Coc; F_(1,26)_ = 123.80, *p* < 0.0001). Bonferroni *post-hoc* test showed the acute psychomotor-stimulating effect of cocaine in each experimental group (Non-CRS/Sal vs. Non-CRS/Coc: ^&^ *p* < 0.05–CRS/Sal vs. CRS/Coc: ^####^ *p* < 0.0001) and significant differences between Non-CRS and CRS after a non-contingent administration of cocaine (Non-CRS/Coc vs. CRS/Coc: **p < 0.01). No differences were found comparing Non-CRS with CRS after a saline i. p. injection (n.s.). **(D)** Schematic timeline of the experimental protocol. **(E,F)** Dopamine levels in the NAcore and NAshell respectively. Baseline measurements were made during 120 min. Saline was administered at 120 min, while cocaine (15 mg/kg i.p.) was injected at 240 min. NAcore: Two-way ANOVA with repeated measures over time revealed significant interaction (Non-CRS vs. CRS x time; F_(12,168)_ = 5.28, *p* < 0.0001), significant effect of stress (Non-CRS vs. CRS; F_(1,14)_ = 24.75, *p* < 0.001) and time (F_(12,168)_ = 8.01, *p* < 0.0001). Bonferroni *post-hoc* test revealed significant differences between Non-CRS and CRS after a cocaine i. p. injection at 270, 330, 390 min (****p < 0.0001) and at 300, 360 min (**p < 0,01). No differences were found comparing Non-CRS with CRS after a saline i. p. injection (n.s.). The effect of cocaine on dopamine efflux was evaluated in each experimental group comparing data at 240 min (last data after a saline i.p. injection) and 270 min (first data in response to a cocaine challenge). Two-tailed unpaired *t*-test revealed the stimulating effect of cocaine on dopamine release (Non-CRS: t_14_ = 2.46; ^&^ *p* < 0.05–CRS: t_14_ = 5.20; ^###^ *p* < 0.001). NAshell: Two-way ANOVA with repeated measures over time showed no interaction (Non-CRS vs. CRS x time; F_(12,132)_ = 1.02, n.s.), no effect of stress (Non-CRS vs. CRS; F_(1,11)_ = 0.09, n.s.), but a significant main effect of time (F_(12,132)_ = 3.46, *p* < 0.001). No differences were found comparing Non-CRS with CRS after a saline i. p. injection (n.s.). The acute effect of a cocaine i. p. injection on dopamine efflux was evaluated in each experimental group comparing data at 240 min and 270 min. Two-tailed unpaired *t*-test revealed the stimulating effect of cocaine on dopamine release (Non-CRS: t_12_ = 2.51; ^&^ *p* < 0.05–CRS: t_10_ = 2.91; ^#^ *p* < 0.05). Baseline dopamine levels were equivalent between non-stressed and stressed animals (NAcore: Non-CRS = 15.04 ± 2.80 fmol/20 µl, CRS = 18.05 ± 2.98 fmol/20 µl; NAshell: Non-CRS = 16.36 ± 3.88 fmol/20 µl, CRS = 24.96 ± 1.64 fmol/20 µl). Two-tailed unpaired *t*-test (Non-CRS vs. CRS) showed non-significant differences between experimental groups either in the NAcore (t_14_ = 0.74; *p* = 0.47) or NAshell (t_11_ = 1.92; *p* = 0.08). **(G)** Illustration of the location of the active membrane of the dialysis probe in the NAcore and NAshell according to Paxinos and Watson (2007). Dashed lines represent probe placements in the Non-CRS group, and solid lines those in the CRS group. All data are shown as mean ± SEM. N is shown in brackets. (CRS = chronic restraint stress, Sal, saline; Coc, cocaine).

Acute or chronic exposure to different types of stressful stimuli influences psychomotor and addictive effects of drugs of abuse as a result of its ability to increase extracellular dopamine in the NA (Sorg and Kalivas, 1993; Deroche et al., 1995; Rouge-Pont et al., 1995; Kalivas and Dufffy, 1998; Kippin et al., 2008; Brodnik et al., 2017). However, the schedule, intensity, and nature of stressful stimuli may have differential effects on dopaminergic activity in the NA (Holly and Miczek, 2016) as well as addiction-related behaviors (Capriles and Cancela, 1999). In this context, we endeavored to determine whether or not CRS has comparable effects to our previous data published after an ARS protocol (Garcia-Keller et al., 2013). In order to characterize the cross-sensitization phenomenon between CRS and cocaine we evaluated behavioral as well as dopamine sensitization in the NA following the experimental design depicted in Figure 1A. Our data showed that a single cocaine i. p. injection of 15 mg/kg was enough to produce behavioral sensitization to cocaine in stress-experienced animals 2 weeks after the last stress session (Figures 1B,C). The saline injection did not modify motor activity in non-stressed or stressed animals meanwhile the psychomotor-stimulating effect of cocaine was observed in both experimental groups (Figures 1B,C).

In another cohort of animals, dopamine levels were examined using the experimental protocol presented in Figure 1D. The results of dopamine monitoring sampled by microdialysis in freely moving rats indicated that a 7-day restraint stress regime induced a greater cocaine-evoked increase of extracellular dopamine in the NAcore, but not the NAshell, than non-stressed animals (Figures 1E,F). No differences between NA subcompartments were found when baseline dopamine levels were evaluated comparing Non-CRS with CRS (NAcore: Non-CRS = 15.04 ± 2.80 fmol/20 µl, CRS = 18.05 ± 2.98 fmol/20 µl; NAshell: Non-CRS = 16.36 ± 3.88 fmol/20 µl, CRS = 24.96 ± 1.64 fmol/20 µl). The saline injection did not modify dopamine release in non-stressed or stressed animals in both NA subcompartments meanwhile the stimulating effect of cocaine on dopamine efflux was revealed in both experimental groups in the NAcore and NAshell (Figures 1E,F). After finishing the microdialysis experiment, the location of the active membrane of each dialysis probe was verified by brain histology as depicted in Figure 1G.

### 3.2. The mitigation of extracellular glutamate levels in response to acute cocaine in the NAcore, but not the NAshell, was consistent with a downregulation of GLT-1 in the NAcore of chronically stressed animals

**FIGURE 2:**
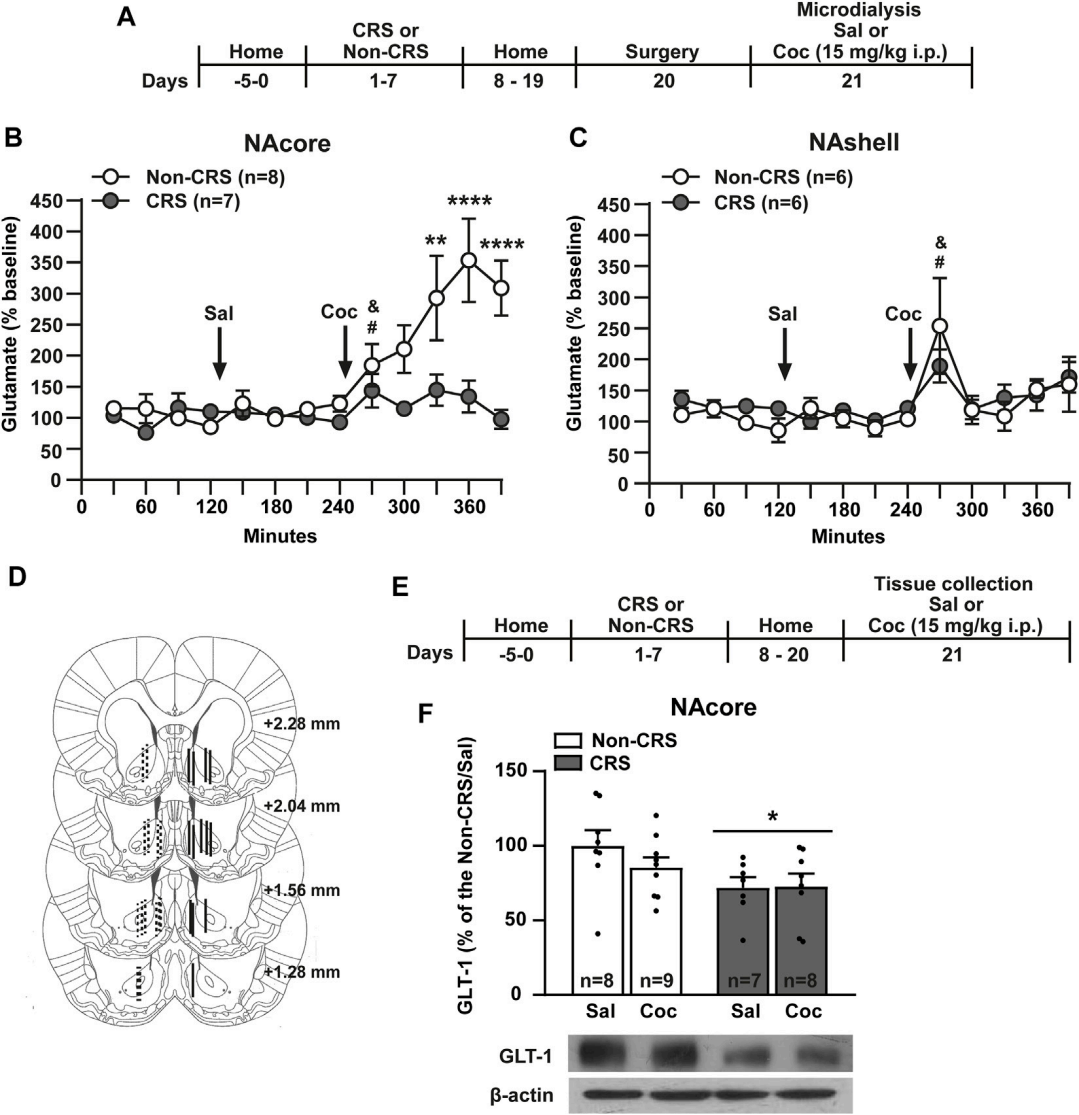
Chronic stress exposure induced a blunted glutamate response after a cocaine i. p. injection and a downregulation of GLT-1 in the NAcore. **(A)** Schematic timeline of the experimental protocol. **(B,C)** Glutamate levels in the NAcore and NAshell respectively. Baseline: measurements were made during 120 min. Saline was administered at 120 min, while cocaine (15 mg/kg i.p.) was injected at 240 min. NAcore: Two-way ANOVA with repeated measures over time showed significant interaction (Non-CRS vs. CRS x time; F_(12,156)_ = 5.04, *p* < 0.0001), significant effect of stress (Non-CRS vs. CRS; F_(1,13)_ = 12.47, *p* < 0.01) and time (F_(12,156)_ = 7.74, *p* < 0.0001). Bonferroni *post-hoc* test revealed significant differences between Non-CRS and CRS after a cocaine i. p. injection at 330 (**p < 0.01), 360 and 390 min (****p < 0.0001). No differences were found comparing Non-CRS with CRS after a saline i. p. injection (n.s.). The effect of cocaine on glutamate efflux was evaluated in each experimental group comparing results at 240 min (last data after a saline i. p. injection) and 270 min (first data in response to a cocaine challenge). Two-tailed unpaired *t*-test revealed an acute effect of cocaine on glutamate release (Non-CRS: t_14_ = 2.29; ^&^ *p* < 0.05–CRS: t_12_ = 2.26; ^#^ *p* < 0.05). NAshell: Two-way ANOVA with repeated measures over time revealed no interaction (Non-CRS vs. CRS x time; F_(12,120)_ = 0.69, n. s.) and no effect of stress (Non-CRS vs. CRS; F_(1,10)_ = 0.29, n.s.) but a significant main effect of time (F_(12,120)_ = 4.41, *p* < 0.0001). No differences were found comparing Non-CRS with CRS after a saline i.p. injection (n.s.). The effect of cocaine on glutamate efflux was evaluated in each experimental group comparing data at 240 min and 270 min. Two-tailed unpaired *t*-test revealed an acute effect of cocaine on glutamate release (Non-CRS: t_10_ = 3.34; ^&^ *p* < 0.05–CRS: t_10_ = 2.45; ^#^ *p* < 0.05). Two-tailed unpaired *t*-test (Non-CRS vs. CRS) revealed that baseline glutamate levels were not significantly different between experimental groups either in the NAcore (t_13_ = 1.74; *p* = 0.11) or NAshell (t_10_ = 0.15; *p* = 0.89). However, in the NAcore, but not the NAshell, a marked trend was observed (Baseline glutamate levels–NAcore: Non-CRS = 24.63 ± 10.36 pmol/10 µl, CRS = 76.29 ± 29.61 pmol/10 µl; NAshell: Non-CRS = 34.86 ± 15.31 pmol/10 µl; CRS = 37.27 ± 5.95 pmol/10 µl). **(D)** Illustration of the location of the active membrane of the dialysis probe in the NAcore and NAshell according to Paxinos and Watson (2007). Dashed lines represent probe placements in the Non-CRS group, and solid lines those in the CRS group. **(E)** Schematic timeline of the experimental protocol. **(F)** GLT-1 protein content was reduced in the NAcore by chronic stress exposure. Two-way ANOVA revealed a significant main effect of stress (Non-CRS vs. CRS; F_(1,28)_ = 5.80, *p < 0.05). There was no significant effect of drug (Sal vs. Coc; F_(1,28)_ = 0.66, n.s.) or interaction (Non-CRS vs. CRS x Sal vs. Coc; F_(1,28)_ = 0.82, n.s.). N is shown in brackets or bars. All data are shown as mean ± SEM. (CRS = chronic restraint stress, Sal, saline; Coc, cocaine).

In line with the above experiments and since a progressive reduction in the increase of glutamate efflux in the stress-related circuitry was observed after repeated exposure to homotypic stressful stimuli (Moghaddam, 2002), we endeavored to determine whether or not CRS has comparable effects to our previous data published after an ARS protocol (Garcia-Keller et al., 2013). In order to explore the ability of cocaine to induce glutamate efflux in the cross-sensitization model, in another cohort of animals, the effect of CRS on glutamate release in response to a cocaine i. p. injection was monitored in the NA, core and shell, through *in vivo* microdialysis following the experimental design shown in Figure 2A. Interestingly, the cocaine-evoked extracellular levels of glutamate in the NAcore were found attenuated in pre-stressed animals compared to non-stressed animals (Figure 2B), an effect that was not observed in the NAshell (Figure 2C). The saline injection did not modify the glutamate levels in non-stressed or stressed animals in both accumbal subcompartments meanwhile cocaine triggered glutamate release in both experimental groups in the NAcore and NAshell (Figures 2B,C). It is important to note that when baseline samples were monitored during the first period of microdyalisis procedure (time-frame from 0 to 120 min), stress-experienced animals tended to exhibit higher baseline levels of extracellular glutamate in the NAcore than non-stressed animals (Non-CRS = 24.63 ± 10.36 pmol/10 µl, CRS = 76.29 ± 29.61 pmol/10 µl). No differences were found between non-stressed and stressed animals when baseline glutamate levels were evaluated in the NAshell (Non-CRS = 34.86 ± 15.31 pmol/10 µl; CRS = 37.27 ± 5.95 pmol/10 µl). After finishing the microdialysis experiment, the probe implantation site was verified by brain histology as depicted in Figure 2D.

Given that GLT-1 is responsible for the uptake of 90%–94% of total extracellular glutamate from synaptic and extra-synaptic sources (Danbolt, 2001), the neurochemical findings observed in the NAcore, but not in the NAshell, could be attributed to an alteration in glutamate transport in this brain region. Thus, the next experiment was conducted to test the GLT-1 expression exclusively in the NAcore, 21 days after the first stress session as shown in Figure 2E. Consistently, our results showed a significant reduction of GLT-1 in the NAcore of CRS-experienced animals compared to those non-stressed (Figure 2F).

### 3.3 CRS increased the density of mushroom-shaped dendritic spines in the NAcore, but not the NAshell

**FIGURE 3:**
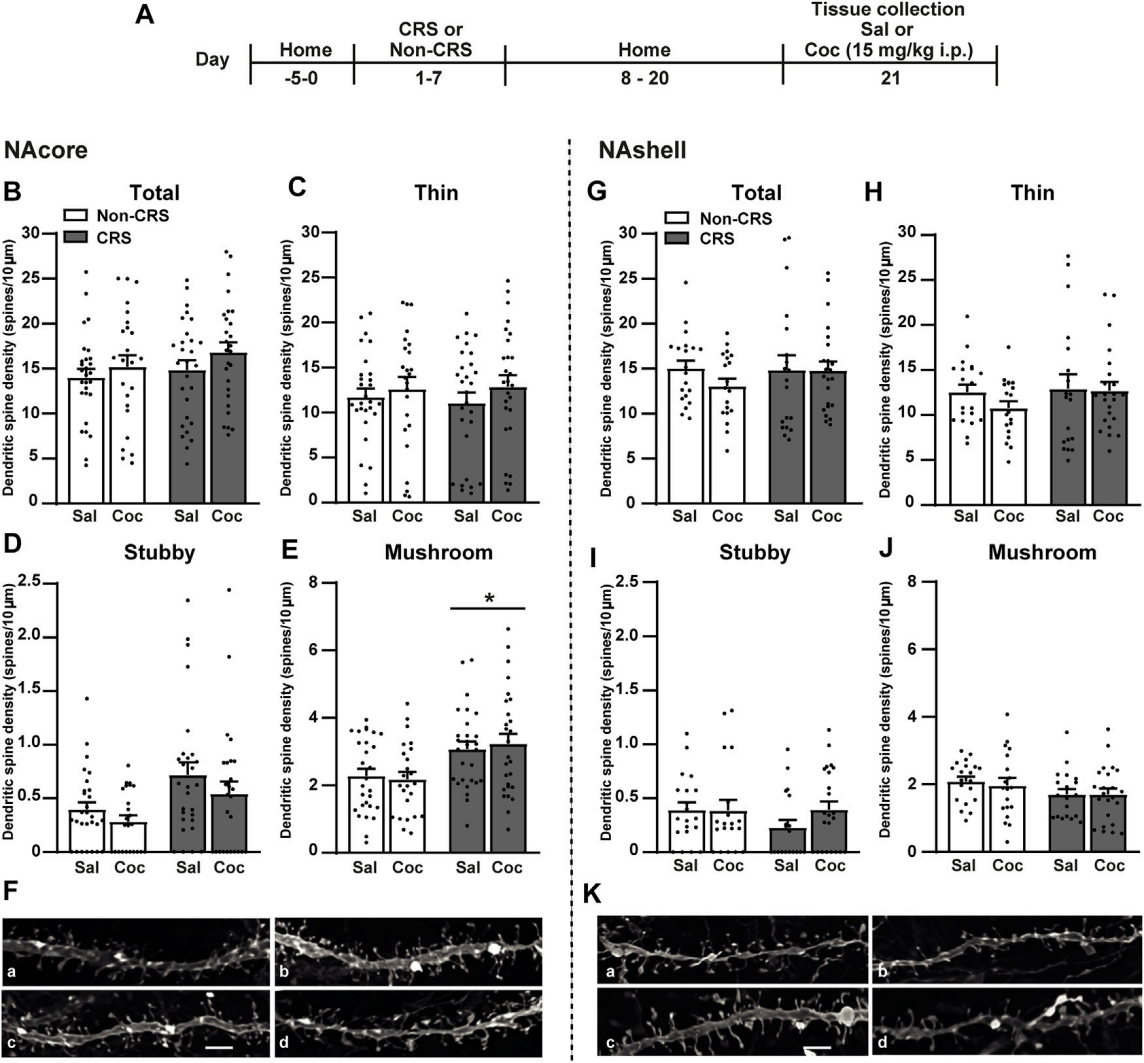
Chronic stress exposure induced an increase in the density of mushroom-shaped dendritic spines exclusively in the NAcore. **(A)** Schematic timeline of the experimental protocol. **(B–E)** Total, thin, stubby and mushroom-shaped dendritic spine density expressed per 10 µm of NAcore MSN dendrite segment. Three to seven segments were analyzed per animal. Total data analyzed: Non-CRS/Sal (28 segments; 1,027 µm dendritic length, seven rats), Non-CRS/Coc (24 segments; 882 µm dendritic length, six rats), CRS/Sal (28 segments; 1,037 µm dendritic length, six rats) and CRS/Coc (27 segments; 823 µm dendritic length, six rats). Total and thin spine density: Two-way nested design ANOVA revealed no effect of stress or drug either for total (Non-CRS vs. CRS; F_(1,22)_ = 0.62, n.s.–Sal vs. Coc; F_(1,22)_ = 0.30, n.s.) or thin spine densities (Non-CRS vs. CRS; F_(1,22)_ = 0.02, n.s.–Sal vs. Coc; F_(1,22)_ = 0.09, n.s.). Although two-way nested design ANOVA revealed interaction for total (Non-CRS vs. CRS x Sal vs. Coc; F_(1,22)_ = 255.23, *p* < 0.0001) and thin spine densities (Non-CRS vs. CRS x Sal vs. Coc; F_(1,22)_ = 118.34, *p* < 0.0001), one-way ANOVA to test significant variations in means among groups showed no statistical differences among them either for total (F_(3,103)_ = 1.12, n.s.) or thin spine densities (F_(3,103)_ = 0.49, n.s.). Stubby spine density: Two-way nested design ANOVA revealed no effect of stress (Non-CRS vs. CRS; F_(1,22)_ = 4.06, n.s.) or drug (Sal vs. Coc; F_(1,22)_ = 0.77, n.s.), but significant interaction (Non-CRS vs. CRS x Sal vs. Coc; F_(1,22)_ = 61.63, *p* < 0.0001). Although one-way ANOVA showed significant variations in means among groups for stubby spine density (F_(3,103)_ = 3.84, *p* < 0.05), Bonferroni *post-hoc* test revealed no significant effect of stress and/or drug (n.s.). Mushroom-shaped spine density: Two-way nested design ANOVA revealed interaction (Non-CRS vs. CRS x Sal vs. Coc; F_(1,22)_ = 230.18, *p* < 0.0001) and a main significant effect of stress (Non-CRS vs. CRS; F_(1,22)_ = 5.33, *p* < 0.05) but non-significant effect of drug (Sal vs. Coc; F_(1,22)_ = 0.24, n.s.). Although one-way ANOVA showed significant variations in means among groups for mushroom-shaped spine density (F_(3,103)_ = 4.91, *p* < 0.01), Bonferroni *post-hoc* test revealed no significant effect of stress and/or drug (n.s.). **(F)** Representative labeling of dendritic shaft and spine images of MSNs in the NAcore: Non-CRS/Sal **(a)**, Non-CRS/Coc **(b)**, CRS/Sal **(c)**, CRS/Coc **(d)**. **(G–J)** Total, thin, stubby and mushroom-shaped dendritic spine density expressed per 10 µm of NAshell MSN dendrite segment. Two to seven segments were analyzed per animal. Total data analyzed: Non-CRS/Sal (20 segments; 835 µm dendritic length, five rats), Non-CRS/Coc (19 segments; 711 µm dendritic length, five rats), CRS/Sal (19 segments; 805 µm dendritic length, five rats) and CRS/Coc (23 segments; 817 µm dendritic length, six rats). Two-way nested design ANOVA revealed no effect of stress or drug either for total (Non-CRS vs. CRS; F_(1,18)_ = 0.02, n.s.–Sal vs. Coc; F_(1,18)_ = 0.34, n.s.), thin (Non-CRS vs. CRS; F_(1,18)_ = 0.13, n.s.–Sal vs. Coc; F_(1,18)_ = 0.44, n.s.), stubby (Non-CRS vs. CRS; F_(1,18)_ = 1.14, n.s.–Sal vs. Coc; F_(1,18)_ = 1.69, n.s.) or mushroom-shaped spine densities (Non-CRS vs. CRS; F_(1,18)_ = 2.513, n.s.–Sal vs. Coc; F_(1,18)_ = 0.001, n.s.). Although two-way nested design ANOVA revealed interaction for total (Non-CRS vs. CRS x Sal vs. Coc; F_(1,18)_ = 212.58, *p* < 0.0001), thin (Non-CRS vs. CRS x Sal vs. Coc; F_(1,18)_ = 172.85, *p* < 0.0001), stubby (Non-CRS vs. CRS x Sal vs. Coc; F_(1,18)_ = 75.87, *p* < 0.0001) and mushroom-shaped spine densities (Non-CRS vs. CRS x Sal vs. Coc; F_(1,18)_ = 282.73, *p* < 0.0001), no significant variations in means among groups were found with one-way ANOVA for total (F_(3,77)_ = 0.64, n.s.), thin (F_(3,77)_ = 0.78, n.s.), stubby (F_(3,77)_ = 1.00, n.s.) and mushroom-shaped spine densities (F_(3,77)_ = 1.20, n.s.). **(K)** Representative labeling of dendritic shaft and spine images of MSNs in the NAshell: Non-CRS/Sal **(a)**, Non-CRS/Coc **(b)**, CRS/Sal **(c)**, CRS/Coc **(d)**. All data are shown as mean ± SEM. Scale bar: 3 μm. (CRS, chronic restraint stress; Sal, saline; Coc, cocaine).

The dynamic shape-changing of dendritic spines by distinct morphological categories (i.e., thin, mushroom, or stubby) provides the notion that synaptic activity changes (Matsuzaki et al., 2001; Kasai et al., 2003, 2010; Holtmaat and Svoboda, 2009; Golden and Russo, 2012). For instance, alterations on glutamate transmission in accumbal circuitry have been associated with enduring structural plasticity of MSNs (Kalivas, 2009). By using the experimental design in Figure 3A, the influence of a 7-day restraint stress regime on dendritic spine morphology was examined. Consistently with data presented here and our previous findings showing a cocaine-triggered increase of GluR1 subunit of AMPA receptors (AMPARs) in the NAcore of CRS-exposed animals (Esparza et al., 2012; Rigoni et al., 2021), an enduring structural remodeling in the NAcore, but not the NAshell, was observed (Figures 3B–K). Specifically, these data revealed non-significant differences among experimental groups for total dendritic spine density (Figure 3B) as well as for thin (Figure 3C) and stubby spine density (Figure 3D) in the NAcore after CRS and cocaine administration. However, an increase in the density of mushroom-shaped dendritic spines (Figure 3E) was observed in pre-stressed animals. In the NAshell, data concerning structural plasticity revealed non-significant differences for total, thin, stubby or mushroom-shaped dendritic spine density (Figures 3G–J).

### 3.4 Ceftriaxone prevented the CRS-induced impairment of glutamate homeostasis in the NAcore as well as behavioral cross-sensitization to cocaine

**FIGURE 4:**
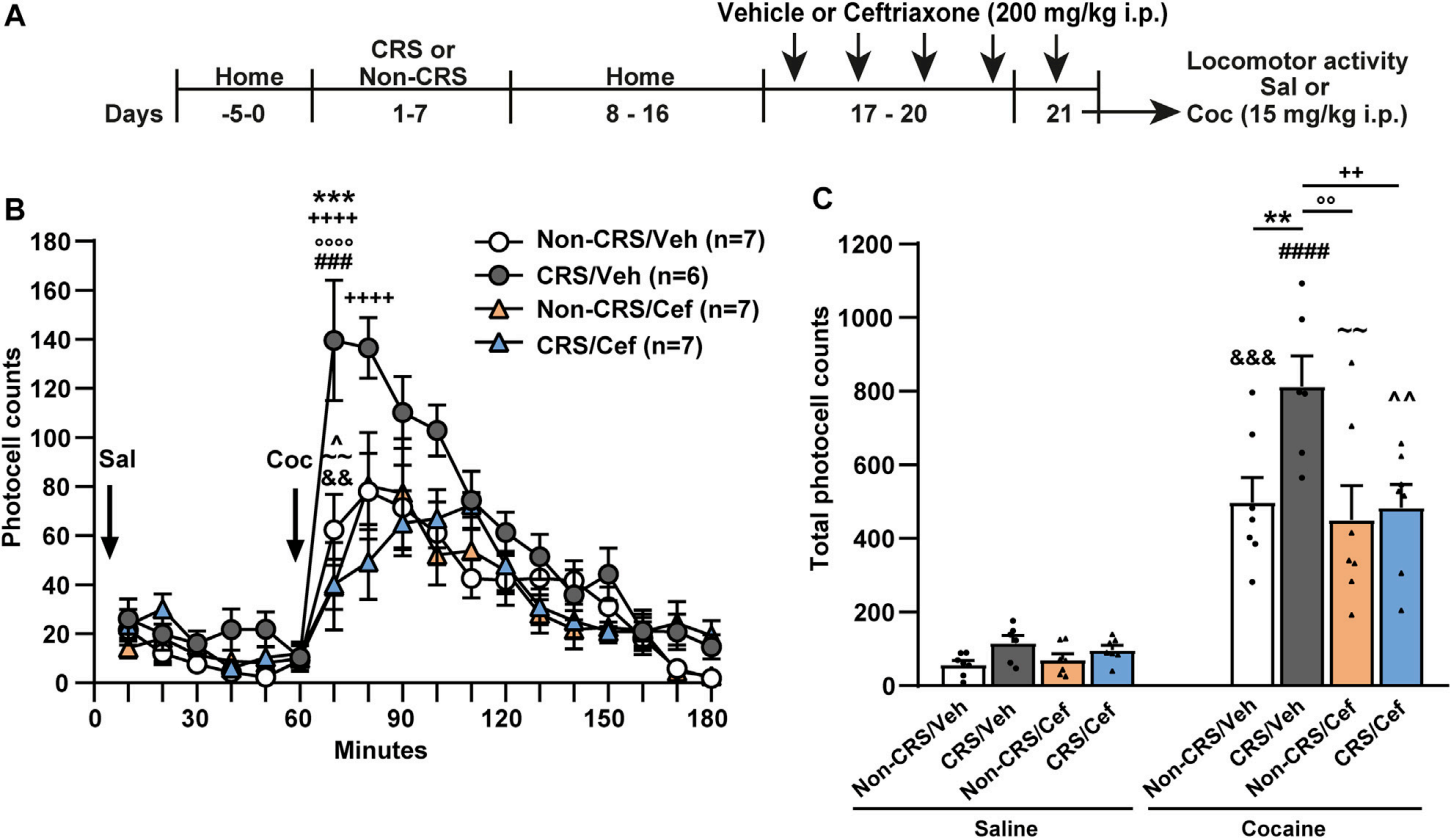
Ceftriaxone prevented the CRS-induced potentiation of locomotor response after acute cocaine administration. **(A)** Schematic timeline of the experimental protocol. **(B)** Time course of the horizontal photocell counts after saline and cocaine (15 mg/kg) i.p. injections. Time-frame from 10 to 60 min corresponds to saline administration at 0 min and time-frame from 70 to 180 min to cocaine administration. Three-way ANOVA with repeated measures over time revealed significant interaction (Non-CRS vs. CRS x Veh vs. Cef x time; F_(17,391)_ = 2.48, *p* < 0.01), significant effect of stress (Non-CRS vs. CRS; F_(1,23)_ = 8.52, *p* < 0.01), ceftriaxone treatment (Veh vs. Cef; F_(1,23)_ = 6.67, *p* < 0.05) and time (F_(17,391)_ = 36.07, *p* < 0.0001). Bonferroni *post-hoc* test revealed significant differences at 70 min (***p < 0.001) when Non-CRS/Veh and CRS/Veh were compared after a cocaine challenge. This multiple comparison test also showed significant differences at 70 and 80 min (^++++^ *p* < 0.0001) when CRS/Veh and CRS/Cef were compared after a cocaine i.p. injection. No differences among experimental groups were found after a saline i.p. injection (n.s.). Ceftriaxone treatment did not evidence a *per se* effect on motor activity as shown comparing Non-CRS/Cef with CRS/Veh at 70 min (°°°°*p* < 0.0001) and Non-CRS/Cef with Non-CRS/Veh at any time (n.s.) in response to a cocaine challenge. No differences were found among experimental groups after a saline i.p. injection (n.s.). The stimulating effect of cocaine on motor activity was evaluated in each experimental group comparing data at 60 min (last data of horizontal motor activity after a saline i. p. injection) and 70 min (first data in response to a cocaine challenge). Two-tailed unpaired *t*-test revealed the psychomotor-stimulating effect of cocaine (Non-CRS/Veh: t_12_ = 3.60; ^&&^ *p* < 0.01—CRS/Veh: t_10_ = 5.11; ^###^ *p* < 0.001—Non-CRS/Cef: t_12_ = 3.13; ^∼∼^ *p* < 0.01—CRS/Cef: t_12_ = 2.50; ^*p* < 0.05). **(C)** Total horizontal photocell counts after a saline or cocaine (15 mg/kg) i.p. injection. Three-way ANOVA indicated non-significant interaction (Non-CRS vs. CRS x Veh vs. Cef x Sal vs. Coc; F_(1,23)_ = 2.20, n.s.), but significant interaction (Non-CRS vs. CRS x Veh vs. Cef; F_(1,23)_ = 4.46, *p* < 0.05), significant effect of stress (Non-CRS vs. CRS; F_(1,23)_ = 8.56, *p* < 0.01), ceftriaxone treatment (Veh vs. Cef; F_(1,23)_ = 6.63, *p* < 0.05) and drug (Sal vs. Coc; F_(1,23)_ = 128.00, *p* < 0.0001). Bonferroni *post-hoc* test showed the acute psychomotor-stimulating effect of cocaine in each experimental group (Non-CRS/Veh/Sal vs. Non-CRS/Veh/Coc: ^&&&^ *p* < 0.001—CRS/Veh/Sal vs. CRS/Veh/Coc: ^####^ *p* < 0.0001—Non-CRS/Cef/Sal vs. Non-CRS/Cef/Coc: ^∼∼^ *p* < 0.01—CRS/Cef/Sal vs. CRS/Cef/Coc: ^^*p* < 0.01), significant differences comparing Non-CRS/Veh/Coc with CRS/Veh/Coc (**p < 0.01) and significant differences comparing CRS/Veh/Coc with CRS/Cef/Coc (^++^ *p* < 0.01). Ceftriaxone treatment did not evidence a *per se* effect on motor activity after a cocaine challenge as shown comparing Non-CRS/Cef/Coc with CRS/Veh/Coc (°°*p* < 0.01) and Non-CRS/Cef/Coc with Non-CRS/Veh/Coc (n.s.). No differences among experimental groups were found after a saline i.p. injection (n.s.). Data are shown as mean ± SEM. N is shown in brackets. (CRS, chronic restraint stress, Veh, vehicle; Cef, ceftriaxone; Sal, saline; Coc, cocaine).

To evaluate the role of GLT-1 in our experimental model, we assessed the effects of systemic ceftriaxone, a known GLT-1 enhancer (Rasmussen et al., 2011), on the CRS-induced behavioral cross-sensitization to cocaine following the protocol presented in Figure 4A. Ceftriaxone abolished the CRS-induced potentiation of the psychomotor-stimulating effect of cocaine. Importantly, this antibiotic did not modify the saline response comparing vehicle-treated with ceftriaxone-treated animals neither the psychomotor-stimulating effect of cocaine comparing ceftriaxone-treated animals after acute cocaine with Non-CRS/Veh/Coc (Figures 4B,C).

**FIGURE 5:**
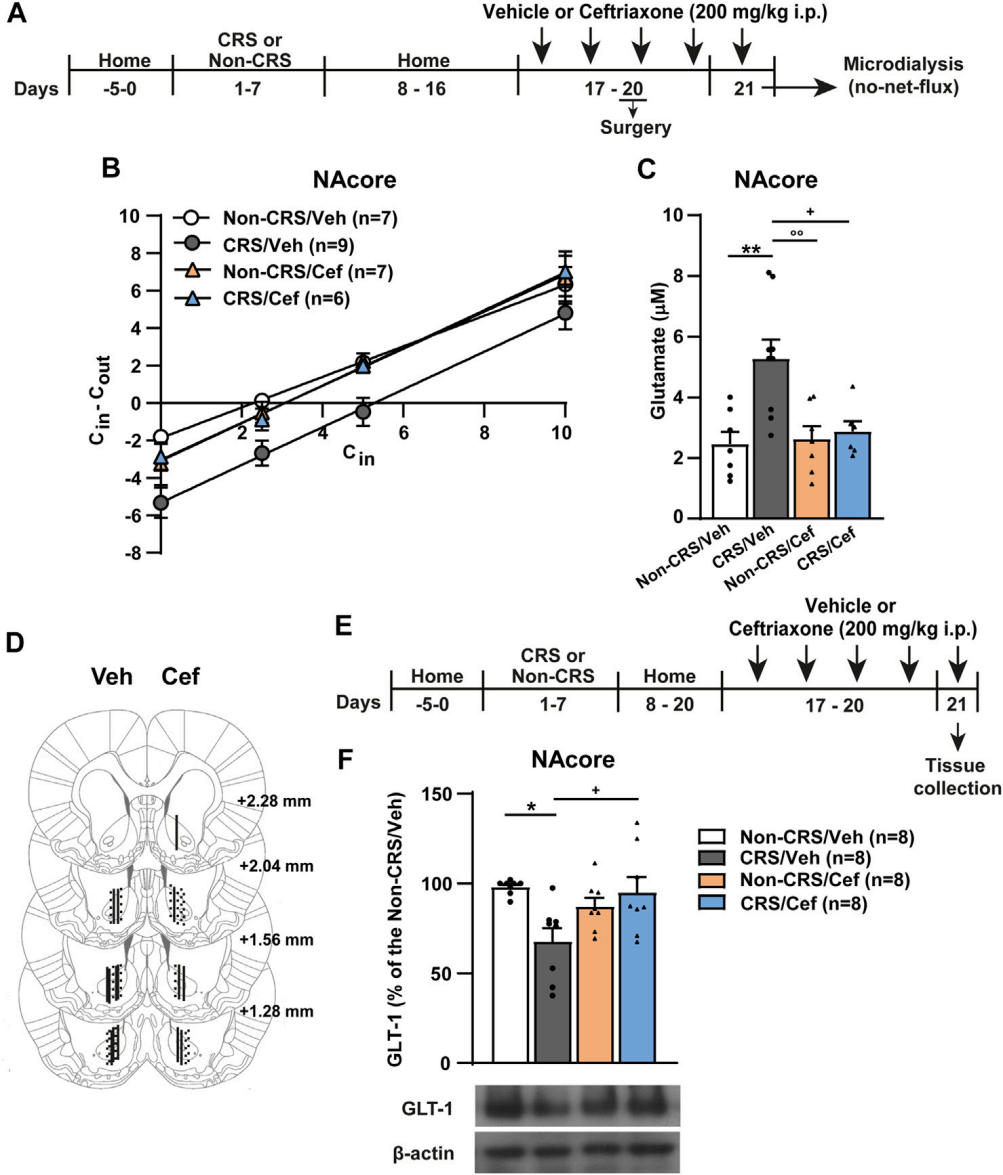
Ceftriaxone restored the CRS-induced GLT-1 downregulation and increased basal concentrations of extracellular glutamate in the NAcore. **(A)** Schematic timeline of the experimental protocol. **(B)** Fitted regression lines of no-net-flux microdialysis in the NAcore (slopes–Non-CRS/Veh: 0.82 ± 0.07, CRS/Veh: 1.01 ± 0.10, Non-CRS/Cef: 0.99 ± 0.12 and CRS/Cef: 1.01 ± 0.08). Linear regression analysis showed non-significant differences among the slopes of the regression line of no-net-flux plot (F_(3,108)_ = 0.83; *p* = 0.48). **(C)** Data obtained from extrapolation from the regression line of each subject belonging each experimental group with *x* axis showed the following basal extracellular glutamate concentrations: Non-CRS/Veh (2.46 ± 0.41) µM, CRS/Veh (5.27 ± 0.63) µM, Non-CRS/Cef (2.62 ± 0.42) µM and CRS/Cef (2.87 ± 0.34) µM. Two-way ANOVA revealed significant interaction (Non-CRS vs. CRS x Veh vs. Cef; F_(1,25)_ = 6.28, *p* < 0.05), significant effect of stress (Non-CRS vs. CRS; F_(1,25)_ = 8.90, *p* < 0.01) and ceftriaxone treatment (Veh vs. Cef; F_(1,25)_ = 4.75, *p* < 0.05). Bonferroni *post-hoc* test showed significant differences comparing Non-CRS/Veh with CRS/Veh (**p < 0.01) and CRS/Veh with CRS/Cef (^+^ *p* < 0.05). Ceftriaxone treatment did not evidence a *per se* effect on basal extracellular glutamate concentrations as shown comparing Non-CRS/Cef with CRS/Veh (°°*p* < 0.01) and Non-CRS/Cef with Non-CRS/Veh (n.s.). **(D)** Illustration of the location of the active membrane of the dialysis probe in the NAcore and NAshell according to Paxinos and Watson (2007). Dashed lines represent probe placements in the Non-CRS group, solid lines those in the CRS group, lines shown in the left hemisphere correspond to the Veh group while lines in the right hemisphere correspond to the Cef group. **(E)** Schematic timeline of the experimental protocol. **(F)** Influence of ceftriaxone treatment on NAcore GLT-1 content. Two-way ANOVA revealed significant interaction (Non-CRS vs. CRS x Veh vs. Cef; F_(1,28)_ = 9.56, *p* < 0.01). There were no significant effects of stress (Non-CRS vs. CRS; F_(1,28)_ = 3.32, n.s.) or treatment (Veh vs. Cef; F_(1,28)_ = 1.81, n.s.). Bonferroni *post-hoc* test showed significant differences comparing Non-CRS/Veh with CRS/Veh (*p < 0.05) and CRS/Veh with CRS/Cef (+*p* < 0.05). Ceftriaxone treatment did not evidence a *per se* effect on basal extracellular glutamate concentrations as shown comparing Non-CRS/Cef with Non-CRS/Veh (n.s.). Data are shown as mean ± SEM. N is shown in brackets. (CRS, chronic restraint stress; Veh, vehicle; Cef, ceftriaxone; Sal, saline; Coc, cocaine).

Since no alterations concerning glutamate efflux after a cocaine challenge and postsynaptic structural remodeling were observed in the NAshell when comparing CRS-exposed animals with Non-CRS animals, we redirected our research to study the NAcore in depth. To clarify the trend toward higher baseline levels of glutamate in the NAcore of pre-stressed animals and to further explain the underlying mechanisms whereby CRS induced impairment of glutamate homeostasis in the NAcore, we focused the next experiment on accurately quantifying the basal concentrations of extracellular glutamate in this brain region through the no-net-flux *in vivo* microdialysis as depicted in Figure 5A. In this experiment, we also included ceftriaxone-treated animals to determine the biological relevance of GLT-1 restoration on glutamatergic mechanisms linked to the altered behavioral response. Basal glutamate concentrations correspond to the *x*-axis interception of the fitted regression line. The slopes of the linear regression plot (Figure 5B) were not different between experimental groups, indicating equivalent *in vivo* probe recovery among groups. Our results confirmed that CRS elicited a lasting increase in basal extracellular glutamate concentrations in the NAcore, an alteration that was prevented by a 5-day ceftriaxone treatment (Figures 5B,C). After finishing the microdialysis experiment, the location of the active membrane of each dialysis probe was verified by brain histology as depicted in Figure 5D. Importantly, when the influence of systemic ceftriaxone treatment on GLT-1 content was determined in animals without experiencing a cocaine challenge and following the experimental design shown in Figure 5E, ceftriaxone due to its ability to enhance GLT-1 levels was able to prevent the CRS-induced GLT-1 downregulation in the NAcore without showing any *per se* effect in non-stressed animals that received ceftriaxone treatment (Figure 5F).

**FIGURE 6:**
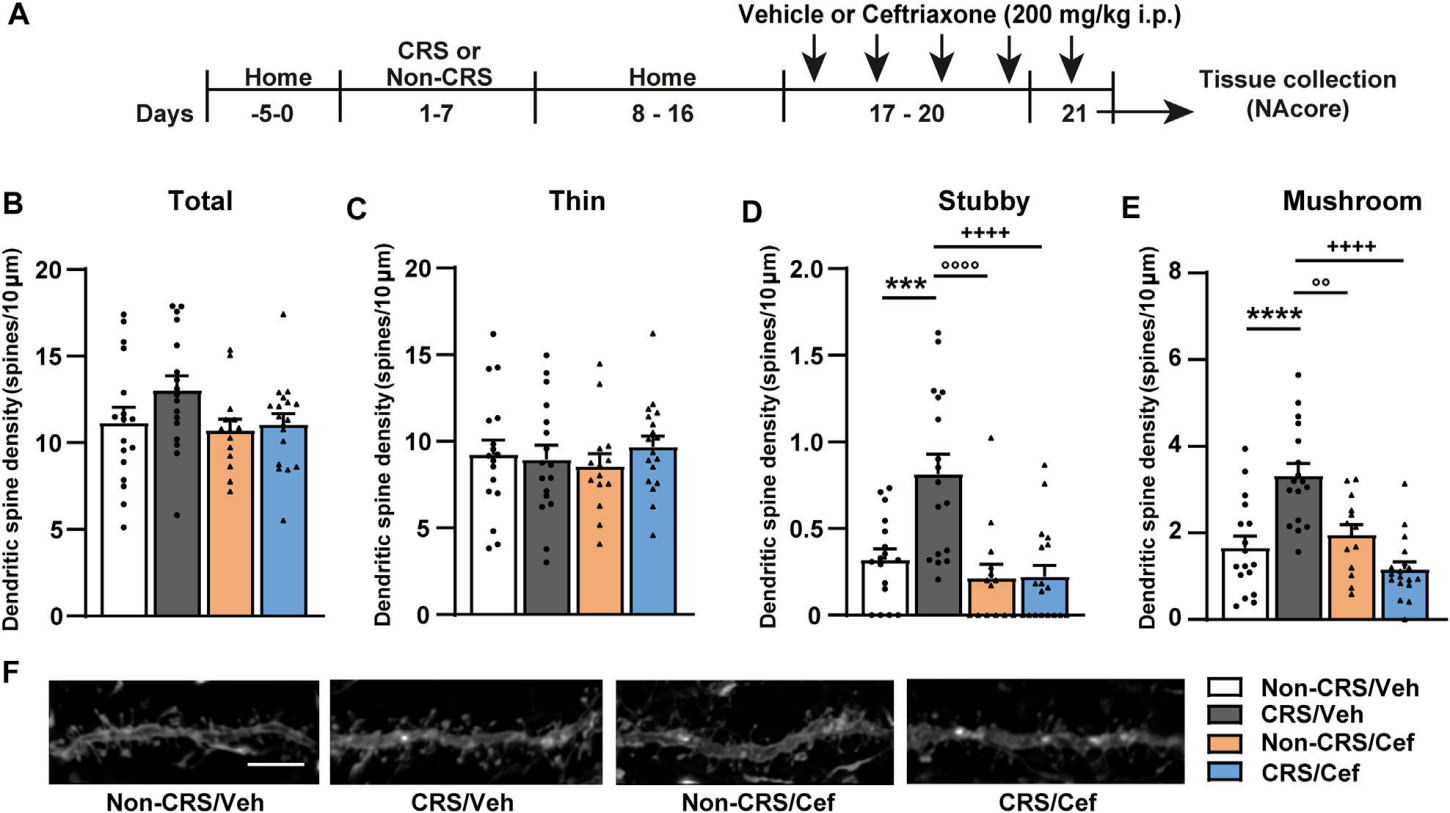
Ceftriaxone prevented the CRS-induced increase in the density of stubby and mushroom-shaped dendritic spines in the NAcore. **(A)** Schematic timeline of the experimental protocol. **(B–E)** Influence of ceftriaxone treatment on total, thin, stubby and mushroom-shaped dendritic spine density expressed per 10 µm of NAcore MSN dendrite segment. Three to six segments were analyzed per animal. Total data analyzed: Non-CRS/Veh (17 segments; 800 µm dendritic length, four rats), CRS/Veh (17 segments; 734 µm dendritic length, four rats), Non-CRS/Cef (14 segments; 600 µm dendritic length, four rats) and CRS/Cef (18 segments; 753 µm dendritic length, four rats). Total and thin spine density: Two-way nested design ANOVA revealed no effect of stress or treatment either for total (Non-CRS vs. CRS; F_(1,13)_ = 0.62, n.s.–Veh vs. Cef; F_(1,13)_ = 2.03, n.s.) or thin spine densities (Non-CRS vs. CRS; F_(1,13)_ = 0.001, n.s.–Veh vs. Cef; F_(1,13)_ = 0.002, n.s.). Although two-way nested design ANOVA revealed interaction for total (Non-CRS vs. CRS x Veh vs. Cef; F_(1,13)_ = 595.85, *p* < 0.0001) and thin spine densities (Non-CRS vs. CRS x Veh vs. Cef; F_(1,13)_ = 271.57, *p* < 0.0001), one-way ANOVA to test significant variations in means among groups showed no statistical differences among them either for total (F_(3,62)_ = 1.93, n.s.) or thin spine densities (F_(3,62)_ = 0.38, n.s.). Stubby spine density: Two-way nested design ANOVA revealed significant interaction (Non-CRS vs. CRS x Veh vs. Cef; F_(1,13)_ = 53.98, *p* < 0.0001), effect of stress (Non-CRS vs. CRS; F_(1,13)_ = 5.78, *p* < 0.05) and treatment (Veh vs. Cef; F_(1,13)_ = 9.50, *p* < 0.01). One-way ANOVA showed significant variations in means among groups for stubby spine density (F_(3,62)_ = 12.06, *p* < 0.0001), Bonferroni *post-hoc* test revealed significant differences comparing Non-CRS/Veh with CRS/Veh (***p < 0.001) and CRS/Veh with CRS/Cef (++++*p* < 0.0001). Ceftriaxone treatment did not evidence a per se effect on stubby spine density as shown comparing Non-CRS/Cef with Non-CRS/Veh (n.s.) and Non-CRS/Cef with CRS/Veh (°°°°*p* < 0.0001). Mushroom-shaped spine density: Two-way nested design ANOVA revealed interaction (Non-CRS vs. CRS x Veh vs. Cef; F_(1,13)_ = 84.16, *p* < 0.0001) and a main significant effect of treatment (Veh vs. Cef; F_(1,13)_ = 5.57, *p* < 0.05) but non-significant effect of stress (Non-CRS vs. CRS; F_(1,13)_ = 1.04, n.s.). One-way ANOVA showed significant variations in means among groups for mushroom-shaped spine density (F_(3,62)_ = 15.79, *p* < 0.0001), Bonferroni *post-hoc* test revealed significant differences comparing Non-CRS/Veh with CRS/Veh (****p < 0.0001) and CRS/Veh with CRS/Cef (++++*p* < 0.0001). Ceftriaxone treatment did not evidence a *per se* effect on stubby spine density as shown comparing Non-CRS/Cef with Non-CRS/Veh (n.s.) and Non-CRS/Cef with CRS/Veh (°°*p* < 0.01). **(F)** Representative labeling of dendritic shaft and spine images of MSNs in the NAcore. All data are shown as mean ± SEM. Scale bar: 5 μm. (CRS, chronic restraint stress; Sal, saline; Coc, cocaine).

Finally, the effect of ceftriaxone on postsynaptic structural remodeling in the NAcore was examined in another cohort of animals without experiencing a cocaine challenge and following the experimental design shown in Figure 6A. Our results revealed that CRS induced an increase in the stubby and mushroom-shaped dendritic spine densities in the NAcore, meanwhile no changes concerning total and thin spine densities were found in CRS-experienced animals (Figures 6B–F). Ceftriaxone restored the CRS-induced structural remodeling in the NAcore (Figures 6D,E).

All these results suggest that the increase of basal extracellular glutamate concentrations via GLT-1 downregulation linked to postsynaptic structural alterations in the NAcore underlies CRS-induced behavioral cross-sensitization to cocaine.

## 4 Discussion

This study showed that a CRS regime is able to induce not only a potentiation of the stimulating properties of cocaine on motor activity and dopamine release in the NAcore, but not the NAshell, but also an impairment of glutamate mechanisms. This disruption of glutamate homeostasis was revealed by blunted glutamate levels in response to cocaine, enhanced basal extracellular glutamate concentrations through GLT-1 downregulation and postsynaptic structural remodeling in the NAcore, alterations that were thought to underlie the CRS-induced cross-sensitization to cocaine. Finally, ceftriaxone, an enhancer of GLT-1, was able to restore the CRS-induced impaired glutamate homeostasis in the NAcore and subsequently prevent stress-induced behavioral alterations. Our findings highlight the biological importance of GLT-1 in the comorbidity between chronic stress exposure and drug abuse.

### 4.1 Potentiation of stimulating properties of cocaine upon motor activity and dopamine release from the NAcore, but not the NAshell, in a CRS-induced cross-sensitization model

Our current data match previous evidence from our laboratory associating ARS-induced behavioral cross-sensitization to cocaine or amphetamine with dopamine sensitization exclusively in the NAcore, in response to a challenge of the drug 21 or 8 days after a single session of stress, respectively (Pacchioni et al., 2007; Garcia-Keller et al., 2013). In line with our results, several studies have reported stress-induced enhancement of psychostimulant effects on locomotor activity and dopamine levels in the NA of stress-experienced animals by foot-shook (Sorg and Kalivas, 1993), food restriction (Deroche et al., 1995; Rouge-Pont et al., 1995), maternal restraint to induce prenatal stress (Kippin et al., 2008) and predator odor (Brodnik et al., 2017) paradigms. Although these latter studies examined the NA, they partly support our data as they do not dissect the involvement of NA subcompartments concerning the influence of stress and addictive drugs on physiological and behavioral mechanisms. Moreover, our data are also consistent with results indicating that stress can activate the mesolimbic dopamine circuit via corticotrophin-releasing factor receptor subtype 1 (CRFR1), which contributes to lasting neural adaptations that amplify the reinforcing and psychomotor-stimulating effects of cocaine (Boyson et al., 2011). Another study focusing on the role of CRFR1 and CRFR2 in the ventral tegmental area (VTA) during an intermittent social defeat stress protocol revealed robust dopamine sensitization in the NAshell as well as stress-induced cross-sensitization to cocaine 10 days after the last stress exposure (Boyson et al., 2014). However, this latter study clearly focused on the examination of dopamine projection from the VTA to the NAshell without analyzing dopamine levels in the NAcore.

With regard to repeated drug exposure-induced behavioral sensitization, this process was related, in animal models of morphine, cocaine and amphetamine sensitization, to increased stimulation of dopamine transmission in the NAcore, but not the NAshell, in response to a drug challenge after 10–15 days of withdrawal (Cadoni and Di Chiara, 1999; Cadoni et al., 2000; Giorgi et al., 2005). Hence, an increase of extracellular dopamine levels in the NAcore appears to be a general feature of the lasting expression of drug- and stress-induced behavioral sensitization to psychostimulants. Interestingly, lasting dopamine sensitization in the NAcore following an amphetamine challenge in pre-stressed animals was reported as a glutamate-dependent mechanism (Pacchioni et al., 2007). This interaction between the dopamine and glutamate systems was proposed as a key event underlying sensitization (Pacchioni et al., 2007; Garcia-Keller et al., 2013). For instance, intra-NAcore infusion of MK-801, an NMDA receptor (NMDAR) antagonist, 30 min before ARS, inhibited stress-induced amphetamine-triggered dopamine and psychomotor sensitization (Pacchioni et al., 2007). A subsequent study demonstrated that the blockade of AMPARs by intra-NAcore infusion of CNQX was able to prevent cocaine-elicited dopaminergic and behavioral sensitization in stress-experienced animals (Garcia-Keller et al., 2013). The persistent glutamate-dependent dopamine enhancement exclusively in the NAcore underlying stress-induced cross-sensitization to psychostimulants reveals: 1) the relevance of this brain region in the enduring proactive effects of stress on vulnerability to drug abuse and, 2) the fact that both neurotransmitter systems constitute common neurobiological substrates to the impact of stress and drugs of abuse.

Although unexplored in our study, given the well-established dopamine-glutamate interaction as the biological substrate that supports the stress-induced vulnerability to drug abuse, it is probable that the cocaine-induced enhancement of dopamine overflow in the NAcore of CRS-experienced animals may be blocked by ceftriaxone. This hypothesis appears to be plausible according to a study revealing that ceftriaxone attenuates the acute cocaine-evoked increase levels of extracellular dopamine in the NA in parallel with a reduced locomotor activity after a cocaine challenge in ceftriaxone-pretreated rats apparently through a GLT-1-independent mechanism (Barr et al., 2015). Taking into account these findings, the ability of ceftriaxone to attenuate the cocaine-induced increase of psychomotor activity observed in our study may be not only by the restoration of glutamate homeostasis but also, in part, due to a reduced dopaminergic neurotransmission in the NAcore.

### 4.2 Relevance of the impairment of glutamate homeostasis in the NAcore, but not the NAshell, to vulnerability to developing cocaine abuse following chronic stress

The disruption of glutamate homeostasis following CRS was revealed as a blunted cocaine-induced increase of extracellular glutamate in the NAcore. This phenomenon was attributed to a tendency to enhance basal extracellular glutamate levels in the NAcore, but not in the NAshell, of CRS-experienced animals. This was then confirmed by using the no-net-flux method. These alterations of glutamatergic mechanisms were related to a decreased expression of GLT-1 in the NAcore. All these results proved to be highly consistent and also matched previous data from our laboratory concerning cross-sensitization between ARS or CRS and cocaine (Avalos et al., 2022; Garcia-Keller et al., 2013, 2016). For instance, resembling dopamine assessment obtained in our laboratory when comparing cocaine-evoked dopamine release after ARS or CRS, a comparable response to that observed after ARS protocol concerning the glutamate efflux following a cocaine i.p. injection in pre-stressed animals (Garcia-Keller et al., 2013) was shown in the NA two weeks after the 7-day restraint stress regime. Likewise, here we found similar results to ARS when we studied basal extracellular glutamate concentrations in the NAcore. Even more, we confirmed that GLT-1 is reduced by 30% in the NAcore long after the CRS regime, a result also consistent with our most recent published data (Avalos et al., 2022). Interestingly, an equivalent downregulated glutamate transport was observed in acutely restrained animals without experiencing a cocaine challenge (Garcia-Keller et al., 2016). As mentioned before, several studies have reported that the repeated exposure to predictable homotypic stressful events is capable of engendering a progressive decrease in the expression of stress responses (Capriles and Cancela, 1999; Moghaddam, 2002; Patel and Hillard, 2008). However, our neurochemical findings concerning glutamate mechanisms and dopamine transmission in the NAcore did not mirror such a habituation phenomenon since a similar neurochemical and behavioral response to that observed after ARS was evidenced (Garcia-Keller et al., 2013, 2016). We hypothesize that the similarity of data observed after CRS and ARS protocols is likely the result of dynamic, but discrete, biological mechanisms capable of buffering the impact of the CRS-induced alterations at molecular, cellular and neurochemical levels preventing a greater response than it has been observed after ARS.

Interestingly, the cocaine-induced enhancement of glutamate release in non-stressed animals constitutes a noteworthy result. Several studies focusing on cocaine sensitization suggest that a prolonged withdrawal period seems to be required for cocaine-induced glutamate neuroplasticity in the NA and the VTA (Pierce et al., 1996; Reid and Berger, 1996; Kalivas and Duffy, 1998). These data are largely in line with our findings observed long after stress exposure. However, it does not explain the increase of extracellular glutamate in control animals after a cocaine challenge in the current study. Many studies have reported a cocaine-evoked increase of glutamate efflux in the NAcore of animals expressing behavioral sensitization to cocaine but not in saline-treated and cocaine non-sensitized rats after 21 days of withdrawal (Pierce et al., 1996). Similar data was revealed after 10 days of withdrawal in cocaine-sensitized rats (Reid and Berger, 1996). Studies in the VTA also demonstrated that a cocaine challenge after 21 days of drug withdrawal increased glutamate release selectively in cocaine-sensitized animals (Kalivas and Duffy, 1998). In contrast to these data but consistently with ours, it was found that regardless of previous amphetamine sensitization or vehicle pretreatment, a high dose of amphetamine induced a delayed increase of glutamate efflux in the NA and the VTA (Xue et al., 1996). Accordingly, it was suggested that high doses of cocaine, likely more than 15 mg/kg, are required to see increased glutamate in drug naïve animals (Smith et al., 1995; Torregrossa and Kalivas, 2008). On the other hand, our current findings are highly reproducible with our previous data published, in which acute cocaine evoked a greater glutamate efflux in the NAcore of animals without experiencing stress (Garcia-Keller et al., 2013). Hence, the fact that a single systemic dose of cocaine (15 mg/kg) may induce a marked rise of extracellular glutamate probably results from a synergistic effect of psychostimulant injection and accumulation of extracellular glutamate because of tissue damage after probe implantation described by Xue et al. (1996).

On the other hand, multiple lines of evidence suggest that, in contrast to the stress-induced alterations in glutamate homeostasis, discontinuing chronic administration of cocaine decreased basal extracellular glutamate levels in the NAcore (Baker et al., 2003; Miguéns et al., 2008) while extracellular glutamate increased in response to a cocaine challenge (Pierce et al., 1996; Reid and Berger, 1996). The mechanisms posited to underlie these findings involve two glial processes induced by cocaine withdrawal: 1) a reduced exchange of cystine-glutamate through the xCT antiporter (Baker et al., 2003; Knackstedt et al., 2010; Trantham-Davidson et al., 2012) and, 2) a reduced glutamate transport via GLT-1 in the NAcore (Knackstedt et al., 2010; Trantham-Davidson et al., 2012).

It is well-established that most of the glutamate sampled by microdialysis arises largely from non-synaptic sources. For instance, glial contribution to dialysis measurements outweighs the synaptic input and provides a dominant role to both cystine–glutamate exchange and glutamate transporter activity (Moussawi et al., 2011). Importantly, these glial mechanisms work together. However, bearing in mind that the cystine-glutamate exchanger works normally by releasing non-vesicular glutamate into the extracellular space, we posit that the changes in extracellular glutamate levels observed following CRS do not depend on alterations in the xCT mechanism but depend strongly on the downregulation of GLT-1. This is supported by the fact that exposure to ARS does not affect the activity of the cystine-glutamate exchanger (Garcia-Keller et al., 2016). Therefore, similarly to ARS, the accumbal disruption of glutamate homeostasis caused by CRS can be attributed to a downregulation of GLT-1 and consequently a reduction in uptake would be expected.

Several studies have related the slope of the linear regression plot of the no-net-flux experiment to neurotransmitter uptake mechanisms. Although there are findings of alterations in GLT-1 expression in parallel with changes in the slope of the no-net-flux plot (Trantham-Davidson et al., 2012; Das et al., 2015), our results did not support such a relationship. There is evidence that differences in slopes reflect a change in the extraction fraction (E_d_) through the microdialysis probe, and this has been empirically demonstrated to be a measure of the clearance of neurotransmitters such as dopamine and acetylcholine (Smith and Justice, 1994; Vinson and Justice, 1997; Bungay et al., 2003). Given that glutamate concentrations are regulated by complex multiple mechanisms (glutamate receptors, transporters and antiporters), the straightforward linkage between slope and E_d_ as an accurate estimation index of uptake may not be the case for glutamate.

The downregulation of GLT-1 in the NAcore is one of the most consistent findings observed after exposure to different classes of drugs of abuse. This alteration, accompanied with a pronounced disruption of glutamate homeostasis in the NAcore, was found following self-administration of cocaine, heroin, alcohol and nicotine (Kalivas, 2009; Knackstedt et al., 2010; Fischer-Smith et al., 2012; Trantham-Davidson et al., 2012; Das et al., 2015; Reissner et al., 2015). Hence, the downregulation of GLT-1 in the NAcore seems to be a feature of vulnerable phenotype to drug-seeking behavior (Kalivas, 2009; Fischer-Smith et al., 2012). Interestingly, the restoring of GLT-1, but not the cystine-glutamate antiporter, through N-acetilcysteine (NAC) treatment (Reissner et al., 2015), as well as the upregulation of GLT-1 induced by ceftriaxone (Sari et al., 2009; Knackstedt et al., 2010; Trantham-Davidson et al., 2012), appeared to be the key mechanisms whereby NAC and ceftriaxone suppress cue-induced cocaine reinstatement. With regard to studies related to the enduring influence of stress on GLT-1 regulation, our results are consistent with findings obtained after different paradigms of stress. For instance, the lasting effect on GLT-1 content and/or function in the hippocampus, cortex and/or striatum was observed in response to inescapable footshock (Almeida et al., 2010; Zink et al., 2010), chronic social defeat stress (Rappeneau et al., 2016) and chronic unpredictable stress (Chen et al., 2014; Liu et al., 2016). Indeed, in line with our data, a study showed a reduced GLT-1 content in the hippocampus and frontal cortex in an experimental model of vulnerability to alcohol intake in animals exposed to early life adverse events (Odeon et al., 2015). The crucial consequences of GLT-1 downregulation underlying ARS- and CRS-induced addictive behaviors were emphasized by two studies from our laboratory which showed that both ceftriaxone and minocycline (this latter through a microglia-dependent mechanism) reversed stress-induced cocaine self-administration and sensitization by restoring GLT-1 in the NAcore (Garcia-Keller et al., 2016; Avalos et al., 2022).

The biological relevance of the CRS-induced downregulation of GLT-1 in the NAcore lies on the fact that this event prevents extracellular glutamate uptake engendering two important consequences: 1) the spillover of synaptic glutamate to the extrasynaptic space that activates perisynaptic mGluR, and 2) the abolished protective effect of GLT-1 on the postsynaptic impact of extracellular glutamate. Accordingly, both mechanisms match our data. First, the fact that the synaptic release of glutamate was found attenuated after a cocaine challenge in the NAcore of pre-stressed animals accounts for glutamate spillover. Under physiological conditions, basal extracellular glutamate, primarily derived from non-synaptic release (i.e., glial source), can regulate glutamate synaptic activity by acting on presynaptic group II metabotropic glutamate receptors (mGluR2/3), which exert an inhibitory tone on the synaptic release of glutamate (Baker et al., 2002). Therefore, any alteration of extracellular glutamate concentration at extrasynaptic level may disturb the synaptic release of glutamate. Since GLT-1 is located close to the synaptic cleft and optimizes glutamate homeostasis (Kalivas, 2009; Scofield et al., 2016), the increase of basal concentrations of extracellular glutamate due to the decreased GLT-1 content could be sensed by mGluR2/3 favoring a rise in the inhibitory tone of these presynaptic metabotropic receptors on the synaptic release of glutamate. As a consequence, this may contribute to the attenuated glutamate efflux when a motivationally important stimulus capable of triggering glutamate efflux is given (e.g., a cocaine challenge). Hence, this mechanism supports the blunted levels of glutamate in the NAcore of pre-stressed animals after a cocaine i. p. injection. Second, the sustained increase of basal extracellular glutamate levels due to the downregulation of GLT-1 may contribute to the enduring postsynaptic structural remodeling long after the CRS regime. In this sense, at the postsynaptic level, an increase in the decay time of NMDA currents was also observed 3 weeks after a single restraint episode. These data supporting glutamate spillover were related to increased basal concentrations of extracellular glutamate driven by GLT-1 downregulation in the NAcore (Garcia-Keller et al., 2013, 2016). Even more, the activation of extrasynaptic NMDARs was abolished after a pharmacological modulation of GLT-1 expression and function, *via* ceftriaxone treatment (Garcia-Keller et al., 2016).

Consistently, here we demonstrated that ceftriaxone restored the basal concentrations of extracellular glutamate in the NAcore in pre-chronically stressed animals, an effect linked to its therapeutic ability as a GLT-1 enhancer. These results strengthen the hypothesis that the normalization of basal glutamate levels may re-establish the tone of presynaptic mGluR2/3, thereby regulating glutamate release after a cocaine challenge as well as postsynaptic structural remodeling, and consequently preventing the expression of CRS-induced behavioral sensitization to cocaine. This substantial mechanism is aligned with that proposed by studies focused on glutamate homeostasis following cocaine self-administration (Moran et al., 2005; Knackstedt et al., 2010; Trantham-Davidson et al., 2012; Logan et al., 2020).

### 4.3 Implications of impaired glutamatergic mechanisms underlying stress-induced addictive behaviors at postsynaptic level

Our findings concerning postsynaptic structural remodeling revealed an increase in the stubby and mushroom-shaped spine densities in the NAcore of CRS-experienced animals. Interestingly, these alterations were rescued by ceftriaxone.

Multiple lines of evidence have reported an increase of stubby spine density in accumbal MSNs 24 h after a social stress regime (Christoffel et al., 2011) specifically in dopamine D2 receptor expressing MSNs (Fox et al., 2020), meanwhile no changes in the mushroom-shaped spine density and a reduced stubby spine density in the central nucleus of the amygdala were shown 14 days after a 14-day restraint stress regime (Moreno-Martinez et al., 2022). In contrast, when mushroom-shaped spines were examined soon after the stress protocol, these studies revealed no changes (Christoffel et al., 2011; Fox et al., 2020) or a marked decrease (Moreno-Martinez et al., 2022). In addition, a selective loss of mushroom-shape spines in anterior bed nuclei of the stria terminalis-projecting cells was observed in animals exposed to 14 sessions of variable stress (Radley et al., 2013). However, these scenarios probably do not mirror ours. First, the time point of measurements is critical in spine dynamics (Kasai et al., 2010), therefore a straightforward comparison between results obtained in these studies and our findings may not be suitable. Second, it was shown that severe stress protocols, such as those employing prolonged stress exposure schedules, may engender dendritic retraction or atrophy with consequences on dendritic spines (Qiao et al., 2016). Indeed, a great heterogeneity of structural changes in dendritic spines depends on the nature of the stressor, intensity of stress exposure, time point of measurement, brain region analyzed and strain (Qiao et al., 2016). For instance, these fluctuations were also observed in animal models of stress and/or substance use disorders (Garcia-Keller et al., 2016; Selvas et al., 2017; McGrath and Briand, 2019; Rigoni et al., 2021; Avalos et al., 2022).

On the other hand, our findings concerning stubby spine density revealed a statistical difference between experiments as shown in Figures 3D, 6D. This difference might be a population issue since two different cohorts of animals were examined. However, it is important to note that both stubby and mushroom spines have been considered large spines (Kasai et al., 2003), whose structures were found to correlate with glutamate sensitivity (Kasai et al., 2003; Matsuzaki et al., 2001, 2004). These studies have reported that large spines, in particular mushroom-shaped spines, exhibited a pronounced glutamate sensitivity (Matsuzaki et al., 2001; Kasai et al., 2003). This observation matches our data since the enduring CRS-induced increase of basal concentrations of extracellular glutamate in the NAcore was accompanied with a persistent CRS-induced increase of mushroom-shaped spines in the same brain region. Hence, it is reasonable to think that the ability of ceftriaxone to rescue such alterations might be attributed to the effective restoration of GLT-1, subsequently removing extracellular glutamate to avoid neurotransmitter spillover susceptible to be sensed by dendritic spines. This idea is also consistent with our previous study (Avalos et al., 2022).

In line with data reported after extended withdrawal from chronic cocaine (Kalivas, 2009; Russo et al., 2010), we posit that our results probably represent a homeostatic compensatory event. Accordingly, the negative modulatory effect of the sustained CRS-induced enhancement of basal extracellular glutamate concentrations on the synaptic release of glutamate may result in the enduring basal increase of mushroom-shaped dendritic spine density as a homeostatic compensation in the NAcore. In addition, after the cocaine challenge there were no significant morphological changes of dendritic spines in pre-stressed animals comparing CRS/Sal with CRS/Coc. Consistently, our previous results indicated a higher surface expression of GluR1 subunit of AMPARs and increased postsynaptic density in accumbal MSNs of pre-stressed animals 45 min after a cocaine challenge, which were related to CRS-induced cocaine behavioral sensitization (Esparza et al., 2012; Rigoni et al., 2021). These robust adaptive plastic alterations, in part, are thought to become a silent synapse in a functional synapse (Dong and Nestler, 2014). These data are aligned with the bidirectional structural change observed in spine head diameter after a non-contingent cocaine challenge to animals withdrawn from chronic cocaine administration. Soon after i. p. injection (45 min), spine head diameter increased in close association with higher levels of AMPARs observed as early as 30 min after acute cocaine. This event was attributed to the increase of extracellular glutamate from synaptic source after a cocaine stimulus (Anderson et al., 2008; Kalivas, 2009). By 120 min, when extracellular glutamate levels were re-established, spine head diameter was significantly reduced, an event that apparently is coupled to a reduction of AMPARs (Boudreau et al., 2007; Kourrich et al., 2007; Kalivas, 2009).

Therefore, altogether these data suggest that while CRS triggers an enduring increase in mushroom-shaped dendritic spines as describe above, the morphological change may not harbor machinery for increasing synaptic strength unless a cocaine injection is administered. This event supports the idea that: 1) mushroom-shaped spines are the structural basis for functional plasticity, and 2) the increase in spine size by stress may be a homeostatic event while increasing synaptic strength is a transient event induced by cocaine. These neuroadaptive changes potentiate glutamate signaling and strengthen glutamate transmission in the NAcore triggering an altered behavioral response.

Last but not least, it is compelling to highlight that our data demonstrated that the impact of stress on dendritic spine morphology was restricted to the NAcore, with no apparent effect of stress or cocaine in the NAshell. This striking divergence between the NAcore and NAshell subcompartments was also seen long after chronic administration of cocaine (Li et al., 2004; Dumitriu et al., 2012). Consistently, electrophysiological changes in the NAcore persisted longer than in the NAshell (Martin et al., 2006) and the long-term increase in spine diameter and/or density remained in the NAcore after weeks of withdrawal from repeated cocaine (Shen et al., 2009) and long after an ARS or CRS regime (Garcia-Keller et al., 2016; Avalos et al., 2022). Importantly, all our findings, including previous published data from our laboratory, are consistent with the fact that the NAcore, but not the NAshell, appears to be the site of long-lasting biological changes, such as neurochemical, neuroplastic and neuroimmune alterations, induced by stress or cocaine that predispose to an addictive phenotype (Garcia-Keller et al., 2013, 2016; De Giovanni et al., 2016; Guzman et al., 2021; Avalos et al., 2022). All these alterations that converge in the impairment of glutamate homeostasis become a straightforward runway to potentiate the rewarding effects of cocaine and facilitate drug abuse or even reinstate drug seeking after long periods of abstinence.

### 4.4 Conclusion

Our findings strongly suggest that the enduring imbalance of the synaptic release of glutamate in response to a cocaine stimulus and the increased basal extracellular glutamate concentrations driven by the CRS-induced downregulation of GLT-1, set the stage for subsequent postsynaptic glutamate adaptations in the NAcore, that do potentiate the effects of cocaine, and may contribute to an enduring vulnerability to the development of cocaine sensitization and addiction. Therefore, the pathophysiological importance of our data lies on the fact that CRS exposure engenders a meaningful print in basal ganglia, surprisingly similar to that observed after ARS, leading a maladaptative response capable of predisposing to drug abuse. Finally, this study points out the biological relevance of GLT-1 as an important comorbidity marker capable of being targeted by pharmacological interventions in the treatment of stress disorders and SUDs and constitutes a sound platform on how exposure to stress increases vulnerability to the development of addiction. Bearing in mind findings that shed light on the epigenetic mechanisms of transgenerational transmission of paternal lifetime experiences (Rodgers and Bale, 2015; Rodgers et al., 2015; Yuan et al., 2016), our study is extremely important in this context since the experience-dependent alterations related to glutamatergic homeostasis thought to underlie addictive behaviors might be communicated across generations and would be capable of shaping neuropsychopathological conditions, including an altered response to stress and SUDs, during early life or adulthood of descendants.

## Data Availability

The original contributions presented in the study are included in the article/Supplementary Material, further inquiries can be directed to the corresponding authors.
