# Supplementary material for: Impairment of glutamate homeostasis in the nucleus accumbens core underpins cross-sensitization to cocaine following chronic restraint stress
Source: Front Physiol. 2022 Aug 26;13:896268. doi: 10.3389/fphys.2022.896268 (PMC9462460; doi:10.3389/fphys.2022.896268)
Supplement: Supplementary file 1 [file DataSheet1.pdf]

SUPPORTING INFORMATION

Impairment of glutamate homeostasis in the nucleus accumbens core underpins cross-sensitization to cocaine following chronic restraint stress

María P. Avalos[a], Andrea S. Guzman[a,†], Constanza Garcia-Keller[a,1,†], Bethania Mongi-Bragato[a], María A. Esparza[a], Daiana Rigoni[a,2,3], Marianela A. Sanchez[a], Gastón D. Calfa[a], Flavia A. Bollati[a*], Liliana M. Cancela[a*]

[a]Instituto de Farmacología Experimental de Córdoba (IFEC-CONICET), Departamento de Farmacología Otto Orsingher, Facultad de Ciencias Químicas, Universidad Nacional de Córdoba, Córdoba, Argentina.

[1]Current address: Department of Neurosciences, Medical University of South Carolina, Charleston, SC, USA.

[2]Current address: Université Côte d'Azur, Nice, France.

[3]Current address: Institut de Pharmacologie Moléculaire et Cellulaire, CNRS UMR 7275, Valbonne, France.

[†]*These authors have contributed equally to this work*

SUPPLEMENTARY EXPERIMENTAL PROCEDURES

1. Microdialysis probe construction

Vertical dialysis probes were prepared in our lab based on previous published data (Garcia-Keller et al., 2013, Pacchioni et al., 2002) and following Di Chiara et al. (1993), with some minor modifications as we described in a recently published work (Avalos et al., 2022; Guzman et al., 2021). Each probe consisted of an inlet and an outlet port and a semipermeable membrane attached and closed at the end of the probe circuit. Probes were constructed using outer 22-gauge stainless steel cannulas connected to fused silica capillary tubings (inner diameter 50 µm, outer diameter 150 µm; Polymicro Technologies, Phoenix, AZ, USA) which extended internally through the probe. The whole assembly was fixed with epoxy cement. The dialysis membrane (AN69HF, Hospal-Gambro, Meyzieu, France) had a 2.0 mm length active dialyzing area and consisted of polyacrylonitrile/sodium methalylsulphonate copolymer (inner diameter 240 µm, outer diameter 310 µm, wall thickness 50 µm, with a molecular weight cutoff of 10 kDa and an average pore size of 29 A). To test the recovery efficiency and the functioning of our microdialysis probes, an *in vitro* recovery assay was performed for all probes used, as described below.

2. In vitro recovery assay for microdialysis probes

For the *in vitro* recovery assay, probes were immersed in Ringer’s solution (NaCl, 145 nM; KCl, 4.0 nM; $CaCl_2$, 2.2 nM; in purified water) containing D-glutamate at a concentration of 40 µM (Sigma Aldrich, USA) while normal Ringer was pumped through the probe at a constant flow rate of 1 µl/min at room temperature (± 22°C). Following 60 min-equilibration time, two successive samples were collected every 30 min and analyzed for glutamate. *In vitro* recovery was calculated by comparing the amount of glutamate present in the sample with the known concentration measured for a sample taken from the solution into which the probe was dipped. The same protocol was used to perform the *in vitro* recovery assay for probes employed in the microdialysis experiment to determine dopamine, except that probes were immersed in Ringer´s solution containing dopamine at a concentration of 12.5 nM (Sigma Aldrich, USA). The *in vitro* recovery percentage for glutamate or dopamine was 14 ± 4 %.

3. Surgery

The day before the microdialysis procedure, rats (280–320 g) were deeply anesthetized with ketamine and xylazine (55 mg/kg and 11 mg/kg, respectively) and mounted into a Stoelting stereotaxic instrument with

the incisor bar at -3.3 cm above the interaural line. Microdialysis probes were then implanted unilaterally in the NAcore (AP = +1.4; ML = +1.6, DV = -7.8) or NAshell (AP = +1.4; ML = +0.8, DV = -7.8) according to coordinates from Paxinos and Watson (2007). The probes were fastened to the skull with glass polyalkenoate (ionomer) cement (Meron; VOCOGmbH, Cuxhaven, Germany), a stainless-steel screw tapped into the skull and dental cement. After surgery, probes were tested with Ringer's solution at flow rate of 0.5 µl/min to check any failure or blockade. All animals were allowed to recover individually in acrylic bowls located in the microdialysis experimental room for 18–22 h. Microdialysis probe placement was verified by brain histology after finishing the microdialysis experiment.

4. Microdialysis and collection of dialysates

The day following surgery (day 21), the microdialysis test began in awake and fully recovered animals. Rats were kept individually in acrylic bowls and provided with free access to food and water during the experiment. The microdialysis probe was connected to a microinfusion pump via FEP Teflon microdialysis tubings. The probes were perfused with Ringer's solution (in nM = NaCl, 145; KCl, 4.0; $CaCl_2$, 2.2) at a constant flow rate of 1 µl/min. Samples of the dialysate were collected every 30 min in vials kept at 4ºC in a refrigerated fraction collector. At the beginning of the experiment, four baseline samples of dopamine or glutamate were collected. Baseline levels were determined when four consecutive samples differed by no more than 10%. Later, all animals received a saline i.p. injection and samples were collected for 120 min. Subsequently, the same rats received cocaine (15 mg/Kg) i.p. injections and, finally, dialysis samples were collected for an additional 150 min. The cocaine dose used in this experiment was the dose we previously mentioned to induce locomotor sensitization. Dialysate samples from individual rats were analyzed for dopamine or glutamate using reverse-phase high-performance liquid chromatography with electrochemical detection (HPLC-EC).

5. Determination of basal concentrations of extracellular glutamate

The basal concentrations of extracellular glutamate were determined through the no-net-flux microdialysis method following our previous data published (Garcia-Keller et al., 2013). The no-net-flux involved adding different concentrations of the analyte of interest (glutamate) to the dialysis buffer. The no-net-flux of the analyte diffusing into or out of the probe was estimated by subtracting the concentration of the analyte added to the dialysis perfusate from the concentration of the analyte in the samples. Thus, to measure basal glutamate concentrations, D-glutamate (Sigma-Aldrich, St Louis, MO,

USA) was added to the dialysis perfusate at concentrations above and below the expected extracellular concentration. Perfusion of Ringer dialysis buffer began in the morning and 2.30 h later, 0, 2.5, 5.0 and 10.0 µM D-glutamate (Sigma-Aldrich, St Louis, MO, USA) was perfused through the probe. Four 30-min dialysis samples were obtained at each concentration of glutamate, and the last three samples were averaged for the determination of the net flux of glutamate. The concentrations of glutamate in the dialysis samples were determined using HPLC-EC as described before. The linear regression plot of the analyte flux at each concentration added to the dialysis buffer yielded the basal level of glutamate (y=0; or the point at which there is no net flux of the analyte into or out of the probe).

6. Histology

At the end of the microdialysis experiments, the animals were decapitated and brains were removed and fixed by the immersion method with a 4% paraformaldehyde (PFA) solution prepared in 0.1 M phosphate buffer. To identify correct placements of the probes, coronal frozen sections (60 µm) were taken using a cryostat (Leica CM1510S, Germany) and mounted on gelatin-coated slides for cresylviolet staining. The histological sections were examined under light microscope to check the position of the probe, which was reconstructed, and positioned referring to the Paxinos and Watson (2007). All animals whose probe traces were found outside the target area were discarded from the statistical analysis.

7. HPLC for dopamine quantification

The perfusate was assayed for dopamine content by reverse-phase HPLC-EC (ESA Coulochem II, Chelmsford, MA, USA) following our previous data published (Garcia-Keller et al., 2013). The mobile phase was composed of (in mM): NaH_2PO_4, 50; Na_2HPO_4, 5; EDTA-Na, 0.1; n-octyl-sodium sulfate, 0.5; and 12% methanol; pH was adjusted to 5.5. The mobile phase was delivered by a pump (Model 582, solvent delivery model; ESA, Chelmsford, MA, USA) at a flow of 1 ml/min through a RP C18 column (Gemini® 3 µm NX-C18 110 Å, 150–4.6 mm, Phenomenex, Torrance, CA, USA). Samples were injected via a 20 µl injection loop. Dopamine was detected using a coulometric detector consisting of three electrodes: a guardcell (+350 mV); an oxidation analytical electrode (+175 mV); and a reduction analytical electrode (+175 mV, analytical cells ESA Model 5014B). Peaks were recorded, and height measured by a computer using an ESA Chromatography Data System (ESA, Inc., Chelmsford, MA, USA). The values obtained were compared with an external standard curve.

8. HPLC for glutamate quantification

The perfusate was assayed for glutamate content by reverse-phase HPLC-EC (ESA Coulochem III, Chelmsford, MA, USA) following our previous data published (Garcia-Keller et al., 2013, Pacchioni et al., 2002). The mobile phase was composed of 100 mM Na_2HPO_4, 1.75% acetonitrile and 20% methanol; pH was adjusted to 6.67 with phosphoric acid. The mobile phase was delivered by a pump (Model 582, solvent delivery model; ESA, Chelmsford, MA, USA) at a flow of 0.6 ml/min through a Waters Xterra MS (15 cm 9 4.6 mm; 3.0 µm). The glutamate released was measured by precolumn derivatization with OPA/OME (o-phthalaldehyde and o-b-mercaptoethanol), as described by Donzanti and Yamamoto (1988). Briefly, 7.5 µl of derivatizing reagent (OPA/OME dissolved in tetraborate buffer) was mixed with 10 µl of the microdialysis eluate. After 2 min of reaction, 10 µl of samples was injected manually into the 20 µl injection loop of the HPLC system. Glutamate was detected using a coulometric detector consisting of three electrodes: a guardcell (+650 mV); an oxidation analytical electrode (+150 mV); and a reduction analytical electrode (+550 mV, analytical cells ESA Model 5014B). Peaks were recorded and the height measured by a computer using an ESA Chromatography Data System (ESA, Inc., Chelmsford, MA, USA). The values obtained were compared with an external standard curve.

9. Membrane Fractionation and Western Blotting for GLT-1

Two weeks after the last stress session (day 21), stressed and non-stressed animals were decapitated 45 min after a saline injection or a cocaine challenge (15 mg/Kg i.p.). Crude membrane fractions were prepared for assessing GLT-1 protein expression. The NAcore was dissected and the bilateral slices were pooled and homogenized in 100 µl ice-cold buffer containing Na Hepes and sucrose (0.32 M sucrose, 1 mM EDTA, 5 mM Tris, pH = 7.4). The buffer was supplemented with 1:100 protease and phosphatase inhibitor mixture. Homogenates were centrifuged at 1000 g for 10 min at 4°C. Next, supernatants were centrifuged at 12000 g for 20 min. The resultant pellet was resuspended in 50 µL radioimmunoprecipitation assay (RIPA) buffer (150 mM NaCl, 0.1% NP40, 0.5% sodium deoxycholate, 0.1% sodium dodecyl sulfate, 50 mM Tris, pH = 7.5) supplemented with protease and phosphatase inhibitors. Samples were combined with appropriate volume of 2x SDS sample buffer (4% SDS, 20% glycerol, 10% b-mercaptoethanol, 125 mM Tris, pH = 6.8) and boiled at 100°C for 5min. Before the addition of 2x SDS sample buffer, aliquots of each sample were used for total protein quantification according to the Bradford method. Western blotting was performed loading 30 µg-protein per lane by 12% SDS-PAGE and then transferring to a PVDF membrane. Membranes were incubated overnight at 4°C with GLT-1 glutamate transporter antibody (1:1000, catalog #3838, Cell Signaling Technology,

Beverly, USA) and then incubated (1 h at room temperature) with peroxidase-conjugated donkey anti-rabbit secondary antibody (1:4000; Jackson Laboratories, Baltimore Pike, PA, USA). Data were normalized to β-actin as loading control and the average of the control group. Membranes were incubated overnight at 4°C with goat anti-Actin polyclonal IgG (1:500, sc 1616, Santa Cruz Biotechnology, Santa Cruz, CA, USA) and then incubated (1 h at room temperature) with peroxidase-conjugated donkey anti-goat secondary antibody (1:2000; Jackson Laboratories, Baltimore Pike, PA, USA).

10. Tissue preparation and DiI labeling

Morphological analysis was performed as previously published (Calfa et al., 2012; Giachero et al., 2015). Briefly, two weeks after the last restraint stress session (day 21) and 45 min after an acute injection of saline or cocaine (15 mg/kg i.p.), animals were deeply anesthetized with ketamine/xylazine (55 mg/kg and 11 mg/kg, respectively) before being perfused transcardially by ice-cold PB (0.1 M, pH 7.4) and fixed using ice-cold 1.5% paraformaldehyde (dissolved in 0.1 M PB, pH = 7.4). Sections were made of the brain containing the area of interest using a vibratome (150 µm thick), and were collected in PBS 0.1 M. Dendritic portions from medium spiny neurons (MSNs) of NAcore and NAshell cells were stained with small droplets (approx. 10 µm) of a saturated solution of the lipophilic dye, 1,1′- dioctadecyl-3,3,3′,3′-tetramethyl indocarbocyanine perchlorate (DiI, Invitrogen, Thermo Fisher Scientific, USA) in fish oil (Giachero et al., 2013; Pozzo-Miller et al., 1999) by microinjection with a patch pipette and positive pressure application. Z-sections from labeled dendritic segments were collected using a laser scanning confocal fluorescence microscope (Olympus FluoView FV1200 or FluoView FV300, Tokyo, Japan) with silicone immersion objective lens (UPlanSApo60x/1.3). The images were deconvolved using the "advanced maximum likelihood estimation algorithm" for Cell R software (version 3.3, Olympus Soft Imaging Solutions). A theoretical point spread function was used. Morphological analysis was performed in Z-stack images as previously published (Calfa et al., 2012; Giachero et al., 2013, 2015; Chapleau et al., 2009; Murphy et al., 1996) using Image J software (National Institutes of Health, USA), and the computer-assisted method, Neuron Studio software (Rodriguez et al., 2008), was also used for spine density and shape evaluation. Single segments from the Z-section projections were used to count the total number and the quantity of each particular type of dendritic spine normalized to 10 µm of the dendritic segment length. Each spine was counted only once by following its course in the Z-section reconstructions. Spine types were classified as thin, stubby and mushroom-shaped dendritic spines, following previously published criteria (Koh et al., 2002; Tyler et al., 2003). Dendritic segments that

belong to different slices from the same rat and from the same experimental group were considered for the statistical analysis.

REFERENCES

Avalos, M.P., Guzman, A.S., Rigoni, D., Gorostiza, E.A., Sanchez, M.A., Mongi-Bragato, B., Garcia-Keller, C., Perassi, E.M., Virgolini, M.B., Peralta-Ramos, J.M., Iribarren, P., Calfa, G.D., Bollati, F.A. and Cancela, L.M. (2022). Minocycline prevents chronic restraint stress-induced vulnerability to developing cocaine self-administration and associated glutamatergic mechanisms: a potential role of microglia. Brain Behav Immun 101, 359-376. https://doi.org/10.1016/j.bbi.2022.01.014

Calfa G, Chapleau CA, Campbell S, Inoue T, Morse SJ, Lubin FD, Pozzo-Miller L. (2012). HDAC activity is required for BDNF to increase quantal neurotransmitter release and dendritic spine density in CA1 pyramidal neurons. *Hippocampus* 22, 1493-1500. https://doi.org/10.1002/hipo.20990

Chapleau CA, Calfa GD, Lane MC, Albertson AJ, Larimore JL, Kudo S, Armstrong DL, Percy AK, Pozzo-Miller L. (2009). Dendritic spine pathologies in hippocampal pyramidal neurons from Rett syndrome brain and after expression of Rett-associated MECP2 mutations. *Neurobiol Dis*35, 219-233. https://doi.org/10.1016/j.nbd.2009.05.001

Di Chiara G, Tanda G, Frau R, Carboni E. (1993). On the preferential release of dopamine in the nucleus accumbens by amphetamine: further evidence obtained by vertically implanted concentric dialysis probes. *Psychopharmacology* 112, 398-402. https://doi.org/10.1007/BF02244939

Donzanti BA, Yamamoto BK (1988). An improved and rapid HPLC- EC method for the isocratic separation of amino acid neurotransmitters from brain tissue and microdialysis perfusates. *Life Sci* 43, 913-922. https://doi.org/10.1016/0024-3205(88)90267-6

Garcia-Keller C, Martinez SA, Esparza MA, Bollati F, Kalivas PW, Cancela LM. (2013). Cross-sensitization between cocaine and acute restraint stress is associated with sensitized dopamine but not glutamate release in the nucleus accumbens. *Eur J Neurosci* 37(6), 982-995. https://doi.org/10.1111/ejn.12121

Giachero M, Calfa GD, Molina VA. (2015) Hippocampal dendritic spines remodeling and fear memory are modulated by GABAergic signaling within the basolateral amygdala complex. *Hippocampus* 25, 545-555. https://doi.org/10.1002/hipo.22409

Giachero M, Calfa GD, Molina VA. (2013). Hippocampal structural plasticity accompanies the resulting contextual fear memory following stress and fear conditioning. *Learn Mem* 20, 611-616. https://doi.org/10.1101/lm.031724.113

Guzman AS, Avalos MP, De Giovanni LN, Euliarte PV, Sanchez, M, Rigoni D, Mongi Bragato B, Virgolini MB, Bollati FA, Cancela LM. (2021). CB1R activation in nucleus accumbens core promotes stress-induced reinstatement of cocaine seeking by elevating extracellular glutamate in a drug-paired context. *Sci Rep* 11, 12964. https://doi.org/10.1038/s41598-021-92389-4

Koh IY, Lindquist WB, Zito K, Nimchinsky EA, Svoboda K. (2002). An image analysis algorithm for dendritic spines. *Neural comput* 14, 1283-1310. https://doi.org/10.1162/089976602753712945

Murphy DD, Segal M. (1996). Regulation of dendritic spine density in cultured rat hippocampal neurons by steroid hormones. *J Neurosci* 16, 4059-4068. https://doi.org/10.1523/JNEUROSCI.16-13-04059.1996

Pacchioni AM, Gioino G, Assis A, Cancela LM. (2002). A single exposure to restraint stress induces behavioral and neurochemical sensitization to stimulating effects of amphetamine: involvement of NMDA receptors. *Ann NY Acad Sci* 965, 233-246. https://doi.org/10.1111/j.1749-6632.2002.tb04165.x

Paxinos G, Watson C. (2007). The Rat Brain in Stereotaxic Coordinates. San Diego: Academic Press/Elsevier.

Pozzo-Miller LD, Inoue T, Murphy DD. (1999). Estradiol increases spine density and NMDA-dependent Ca2+ transients in spines of CA1 pyramidal neurons from hippocampal slices. *JNeurophysiol* 81, 1404-1411. https://doi.org/10.1152/jn.1999.81.3.1404

Rodriguez A, Ehlenberger DB, Dickstein DL, Hof PR, Wearne SL. (2008). Automated three-dimensional detection and shape classification of dendritic spines from fluorescence microscopy images. *PloS One* 3:e1997.12. https://doi.org/10.1371/journal.pone.0001997

Tyler WJ, Pozzo-Miller L. (2003). Miniature synaptic transmission and BDNF modulate dendritic spine growth and form in rat CA1 neurones. *J Physiol* 553, 497-509. https://doi.org/10.1113/jphysiol.2003.052639
